# HIV acquisition in pregnancy: implications for mother‐to‐child transmission at the population level in sub‐Saharan Africa

**DOI:** 10.1002/jia2.25783

**Published:** 2021-09-21

**Authors:** Milly Marston, Kathryn Risher, Mary I. Mahy

**Affiliations:** ^1^ Department of Population Health London Schoolof Hygiene and Tropical Medicine London UK; ^2^ UNAIDS Geneva Switzerland

**Keywords:** HIV, HIV acquisition, paediatric, pregnancy, postpartum, vertical transmission

## Abstract

**Introduction:**

A recent sero‐discordant couple study showed an elevated risk of HIV‐acquisition during the pregnancy/postpartum period per‐condomless‐coital‐act. This, along with previous studies, has led to concern over possible increased risk of mother‐to‐child (vertical) transmission, due to the initial high viral load in the first months after seroconversion, in a time when the woman and health services may be unaware of her status. This study looks at whether behavioural differences during the pregnant/postpartum period could reduce the impact of elevated risk of HIV acquisition per‐condomless‐coital‐act at the population level.

**Methods:**

We used data from 60 demographic and health surveys from 32 sub‐Saharan African countries. Using the HIV status of couples, we estimated differences in serodiscordancy between HIV‐negative women who were pregnant/postpartum compared to those who were not pregnant/postpartum. We compare the risk of sexual activity over the pregnant/postpartum period to those not pregnant/postpartum. Using these risks of serodiscordancy and sexual activity along with estimates of increased HIV risk in the pregnancy/postpartum period per‐condomless‐coital‐act, we estimated a population‐level risk of HIV acquisition and acute infection, during pregnancy/postpartum compared to those not pregnant/postpartum.

**Results:**

Sexual activity during pregnancy/postpartum varies considerably. In general, sexual activity is high in the first trimester of pregnancy, then declines to levels lower than among women not pregnant/postpartum, and is at its lowest in the first months postpartum. Adjusted for age and survey, pooled results show HIV‐negative pregnant women are less likely to have an HIV‐positive partner compared to those not pregnant/postpartum (risk ratio (RR) = 0.78, 95% CI = 0.68–0.89) and comparing the postpartum period (RR = 0.85, 95% CI = 0.73–0.99). Estimated population‐level risk for HIV acquisition and acute infection in pregnancy/postpartum was lower than would be inferred directly from per‐condomless‐coital‐act estimates in most countries, over the time of most risk of mother‐to‐child transmission, though there was variation by country and month of pregnancy/postpartum.

**Conclusions:**

Estimates of population‐level HIV acquisition risk in sub‐Saharan Africa should not be taken directly from per‐condomless‐coital‐act studies to estimate vertical transmission. Changes in sexual behaviour and differences in HIV‐serodiscordancy during pregnancy/postpartum reduce the impact of increased risk of HIV acquisition per‐condomless‐coital‐act, this will vary by region.

## 1 INTRODUCTION

A recent study of sero‐discordant couples provides strong evidence for an elevated risk of HIV acquisition per‐condomless‐coital‐act during pregnancy and the postpartum period [1]. This finding, along with previous findings supporting an increased risk of HIV acquisition in pregnancy [2, 3], has led to concern over the possible higher risk of mother‐to‐child (vertical) transmission due to the initial high viral load seen in the first months after seroconversion [4, 5], during a time when the woman and health services may be unaware of her status.

A recent systematic literature review and meta‐analysis [6] found high HIV incidence during pregnancy/postpartum, but in studies comparing pregnant/postpartum women to those not pregnant/postpartum, the average hazard ratios showed no evidence for an increased risk in HIV acquisition in either period, though substantial heterogeneity was observed across the studies.

Interpreting results on HIV acquisition in pregnancy and the postpartum period is clearer with per‐condomless‐coital‐act‐studies [1], which attempt to look at the purely biological aspect of HIV acquisition, compared to general population studies [7, 8], which look at population‐level HIV acquisition as a result of biology and behaviour. These two types of studies give very different results. Thomson et al. [1] found a much higher risk of HIV acquisition per‐condomless‐coital‐act in pregnant women in late pregnancy and in the 6 months postpartum compared to those not pregnant/postpartum. Marston et al. [8], using population‐based data, found after adjusting for the age that there was no difference in HIV acquisition during the pregnant/postpartum period compared to those not pregnant/postpartum.

The rate of HIV acquisition and differences between pregnant/postpartum and not pregnant/postpartum women at a population level will depend not only on the risk of infection per‐condomless‐coital‐act with an HIV‐positive partner, but also on differences in the level of serodiscordance and in sexual behaviours between these groups [1, 9]. Therefore, acquisition rates from sero‐discordant studies per‐coital‐act cannot be generalised as population‐level risks for women, which are necessary to model national estimates of paediatric HIV [10].

This study aims to understand the implications of an increased risk of HIV acquisition per‐condomless‐coital‐act during pregnancy and postpartum on population‐level estimates of vertical transmission. We used demographic and health surveys (DHS) to investigate differences in partnership serodiscordancy and different levels of sexual activity by months since conception. Using these analyses and estimates from published literature, we created estimates of the population risk of HIV acquisition and acute HIV‐infection in the pregnant/postpartum period compared to periods when women are not pregnant or postpartum, given per‐condomless‐coital‐act transmission rates.

## 2 Methods

### 2.1 Data

We used data on women aged 15–49 from 60 nationally representative DHS and AIDS indicator surveys (AIS) conducted in 32 sub‐Saharan African countries between 2003 and 2017 in which HIV testing outcomes were available (Table 1) [11]. From these, 53 surveys from 29 countries were available with couples linking and HIV status data (Table 1). See Appendix S1.

**Table 1:** Availability of surveys in sub‐Saharan Africa and the unweighted number of HIV‐negative women overall and in the couple sample

Region	Survey country and year	HIV‐negative women Unweighted N	HIV‐negative women in couple data Unweighted N
Southern and eastern Africa	Angola, 2015–16	6427	2250
	Eswatini, 2006–07	3146	460
	Ethiopia, 2005	5800	2617
	Ethiopia, 2010–11	15,147	6376
	Ethiopia, 2016	14,165	5790
	Kenya, 2003	2996	1109
	Kenya, 2008–09	3493	1225
	Lesotho, 2004–05	2232	498
	Lesotho, 2009–10	2852	655
	Lesotho, 2014	2384	529
	Malawi, 2004–05	2443	1266
	Malawi, 2010	6506	3218
	Malawi, 2015–16	6860	3228
	Mozambique, 2009	8988	–
	Mozambique, 2015	5647	2190
	Namibia, 2013	4170	979
	Rwanda, 2005	5441	2102
	Rwanda, 2010–11	6686	2723
	Rwanda, 2014–15	6495	2792
	South Africa, 2016	1955	321
	United Republic of Tanzania, 2003–04	10,048	–
	United Republic of Tanzania, 2007–08	14,437	–
	United Republic of Tanzania, 2011–12	16,930	–
	Uganda, 2011	19,871	–
	Zambia, 2007	4766	2182
	Zambia, 2013–14	13,105	5961
	Zimbabwe, 2005–06	5941	1760
	Zimbabwe, 2010–11	6389	2205
	Zimbabwe, 2015	7471	2710
Western and central Africa	Burkina Faso, 2003	4105	2217
	Burkina Faso, 2010	8246	4961
	Burundi, 2010–11	4401	1924
	Burundi, 2016–17	8373	3513
	Cameroon, 2004	4805	1948
	Cameroon, 2011	6819	2755
	Chad, 2014–15	5696	2874
	Congo Democratic Republic, 2007	4551	2219
	Congo Democratic Republic, 2013–14	9183	4359
	Congo, 2009	11,735	–
	Cote d'Ivoire, 2005	8078	–
	Cote d'Ivoire, 2011–12	4446	1877
	Gabon, 2012	5174	1800
	Gambia, 2013	4394	1300
	Ghana, 2003	5151	1957
	Ghana, 2014	4568	1747
	Guinea, 2005	3774	1883
	Guinea, 2012	4581	2181
	Liberia, 2006–07	6335	2422
	Liberia, 2013	4294	1720
	Mali, 2006	4674	2572
	Mali, 2012–13	5044	2868
	Niger, 2006	4402	2128
	Niger, 2012	5074	2656
	Sao Tome and Principe, 2008–09	2513	904
	Senegal, 2005	4417	1297
	Senegal, 2010–11	5529	1704
	Senegal, 2017	7909	2465
	Sierra Leone, 2008	3402	1619
	Sierra Leone, 2013	7724	3530
	Togo, 2013–14	4680	2197

### 2.2 Analysis

#### 2.2.1 Definitions of pregnancy and the postpartum period

The risk of mother‐to‐child transmission occurs in the period of pregnancy, delivery and during breastfeeding in the postpartum period. Using self‐reported pregnancy information, date of birth of the last child and whether a woman was currently breastfeeding, we created a maternity status variable “months since conception” with months 1–9 being pregnancy and months 10–15 representing up to 6 months postpartum breastfeeding. Further details are given in Appendix S1.

#### 2.2.2 Differences in couple serodiscordancy by months since conception

HIV‐negative women were grouped into having an HIV‐positive partner or not, using surveys where couple linking was available. Due to the smaller size of the couple sample, we used the months since conception variable grouped into 3‐month periods. Other variables used were 5‐year age group, country and survey year. With the pooled data, we used log‐binomial regression to calculate the risk ratios of an HIV‐negative woman having an HIV‐positive partner, comparing each trimester of pregnancy and early and late postpartum women to those not pregnant/postpartum, adjusting for 5‐year age group, country and year. From these risk ratios, we interpolated monthly risk ratios. Further details are available in Appendix S1.

#### 2.2.3 Sexual activity by months since conception

To assess the difference in sexual activity by months since conception, we used HIV‐negative women aged 15–49. Condomless sexual activity was defined as reporting sexual intercourse in the last week prior to the survey and not reporting current condom use, either as a current contraceptive method or with the last sexual partner. We assumed that sexual activity in the last week had the same relationship to coital frequency over all months since conception. We used log‐binomial regression to calculate the risk ratios of reporting recent condomless sex, comparing each month of pregnancy/postpartum to those not pregnant/postpartum, adjusting for 5‐year age group, place of residence and calendar year for each country where data were available. We also looked at whether there was evidence of an interaction between the co‐variates and months since conception.

#### 2.2.4 HIV acquisition per‐condomless‐coital‐act risk by months since conception

We used the adjusted risk ratios of HIV acquisition per‐condomless‐coital‐act among women with HIV‐positive male partners by months since conception from Thomson et al. [1]. These are given for three periods: early pregnancy, defined as the first 3 months; late pregnancy, 4 to 9 months pregnant; and postpartum, the 24 weeks after the end of pregnancy. Thomson et al. [1] showed changes in the risk ratio over early and late pregnancy and discussed that these may be due to high levels of oestrogen and progesterone. Since these two hormones increase throughout pregnancy [12], we interpolated risk ratios for each month of pregnancy assuming a linear change. The risk ratio for each period was fitted at the midpoint of the early and late pregnancy periods, with each period's mean risk ratio equal to that in Thomson et al. (using a geometric progression [1]). There is only one risk ratio given for the postpartum period in Thomson et al. Reasons given for the higher risk of HIV acquisition in this time are both from hormonal shifts and the presence of macro‐ and micro‐trauma caused by the delivery or vaginal dryness caused by low oestrogen during breastfeeding [1]. Since trauma from birth is most acute in the first weeks after the birth, it is reasonable to think that the risk at this time might be higher than later in the postpartum period, supported with some evidence in a study of HIV incidence during breastfeeding in South Africa [13]. We chose a gradient that fitted this but also assessed different gradients to see how results might vary.

### 2.3 Other inputs

This analysis focuses on the acute infection window, where there is a higher risk of vertical transmission. For this, we need to consider first when vertical transmission occurs and the length of the acute infection window, the vast majority of vertical transmission is most likely to occur in the last month of pregnancy, intrapartum or postpartum during breastfeeding [14, 15]. Therefore, when considering the impact of HIV acquisition on vertical transmission in relation to modelling of the epidemic, we only consider that vertical transmission occurs in the last month of pregnancy and the postpartum breastfeeding period. We used an acute infection window of 3 months; this assumption is likely to be an upper estimate [5, 16, 17]. We used country‐specific female incidence rates from UNAIDS estimates for the year 2019 (Appendix S1).

#### 2.3.1 Estimating population‐level HIV acquisition risk ratios and acute infection ratios

Using the inputs described above, we estimated the population‐level HIV acquisition risk ratio for pregnant/postpartum women compared to those not pregnant/postpartum, for each month of the period, as a product of the risk ratios for the per‐condomless‐coital‐act risk of HIV acquisition from Thomson et al. [1], risk of condomless sexual activity and risk of having an HIV‐positive partner. We calculated two life tables, one for women moving through pregnancy/postpartum and one for women not pregnant/postpartum, to estimate the number of new infections that we would expect in each month. From this, we estimated the number of acute infections for each month by summing the new infections of that month and the 3 months before, since new infections will remain acute for 3 months. From these monthly acute infections, we can create a ratio of acute infections for each month since conception comparing pregnant/postpartum women to those not pregnant/postpartum. We assume no antiretroviral therapy (ART) initiation. We performed a number of sensitivity analyses to assess the impact of changing the assumptions underlying our estimated population‐level HIV acquisition risk ratios.

### 2.4 Ethics

This analysis uses secondary data from the DHS, which are freely available upon request. Standard DHS surveys have been reviewed and approved by the ICF Institutional Review Board (IRB) [11]. Additionally, country‐specific DHS survey protocols are reviewed by the ICF IRB and typically by an IRB in the host country. Informed consent was gained for the surveys and HIV testing. Ethical approval for this study was obtained from the London School of Hygiene and Tropical Medicine ethics committee.

## 3 Results

### 3.1 Serodiscordancy by months since conception

In the pooled model across all countries, there was strong evidence of a lower risk of having an HIV‐positive partner when pregnant and postpartum (Table 2). For pregnancy, the risk ratio decreased each trimester, with pregnant women in the third trimester having 0.69 (95% CI = 0.55–0.97) times the risk of not pregnant/postpartum women. In the early postpartum period, HIV‐negative women had 0.73 (95% CI = 0.62–0.86) times the risk of having an HIV‐positive partner compared to those not pregnant/postpartum. The late postpartum period showed no evidence for a difference in risk between postpartum women and those not pregnant/postpartum (RR = 0.95, 95% CI = 0.79–1.14). There was no significant interaction between months since conception and 5‐year age group (Wald test *F* = 060, *p* = 0.944). There was no evidence for a difference by region. Similar results were found restricting those not reporting condom uses (Appendix S1). The proportion of all women who contributed to this analysis, varied by months since conception and country (Appendix S1).

**Table 2:** Pooled adjusted risk ratio for being in a sero‐discordant relationship, HIV‐negative women by months since conception compared to not pregnant/postpartum

	Month of pregnancy/postpartum period	Three months aRR^*^ from regression models	Monthly RR
Pregnancy	1st month pregnant	0.89 (0.70–1.14)	0.94
	2nd month pregnant		0.89
	3rd month pregnant		0.84
	4th month pregnant	0.76 (0.62–0.93)	0.80
	5th month pregnant		0.76
	6th month pregnant		0.74
	7th month pregnant	0.69 (0.55–0.87)	0.71
	8th month pregnant		0.69
	9th month pregnant		0.67
Postpartum	1st month postpartum	0.73 (0.58–0.91)	0.67
	2nd month postpartum		0.73
	3rd month postpartum		0.80
	4th month postpartum	0.95 (0.79–1.14)	0.87
	5th month postpartum		0.95
	6th month postpartum		1.04

^*^Adjusted for 5‐year age group, calendar year and country.

Monthly RRs represent interpolated monthly risk ratios. aRR, adjusted risk ratio; RR, risk ratio.

### 3.2 Sexual activity by months since conception

**Figure 1:**
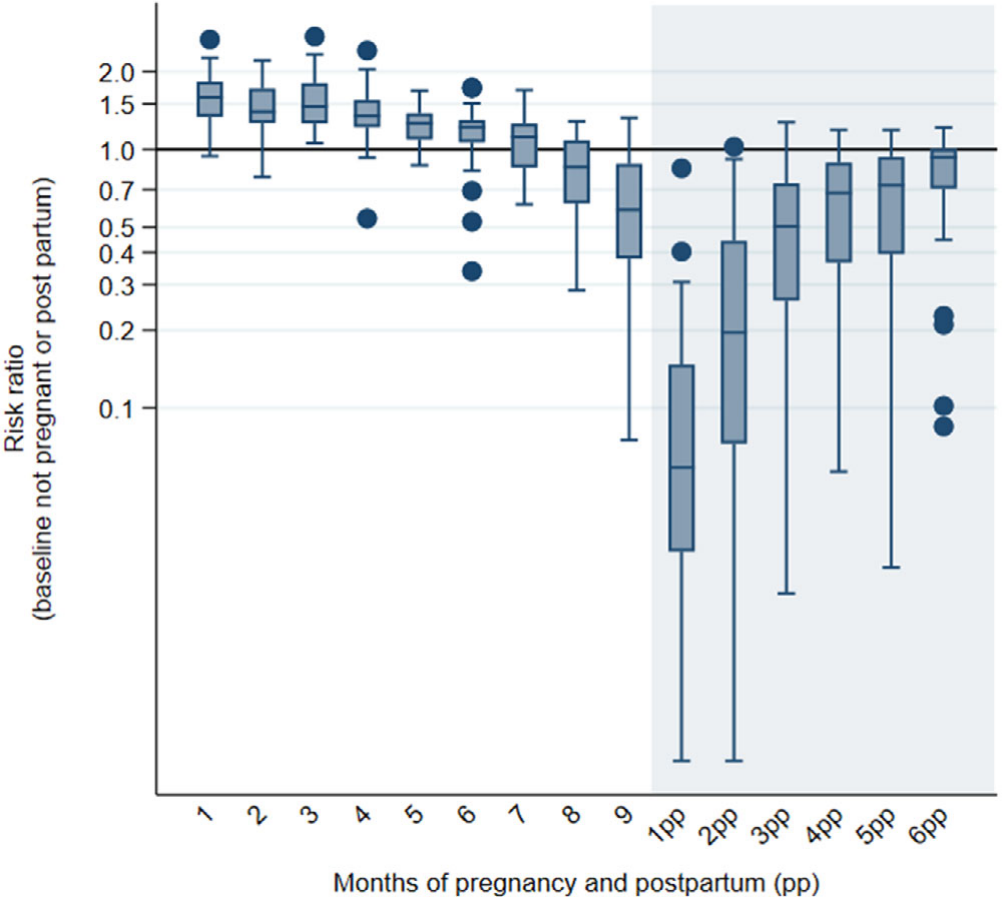
Risk ratios of sexual activity in the last week during the pregnant and postpartum period compared to not pregnant/postpartum, HIV‐negative women, aged 15–49, adjusted for 5‐year age group and calendar year for 29 countries.

Sexual activity by months since conception varied greatly by country. However, there was a similar pattern in all countries when comparing to those not pregnant/postpartum. The overall pattern and country variation are shown in Figure 1. In the first trimester, reported sexual intercourse in the last week is higher than that outside the pregnancy and postpartum periods, it then slowly reduces through pregnancy with a steep decrease in the last trimester, often to levels lower than that outside the pregnancy/postpartum periods. In the first 2 months of the postpartum period, there was a much lower risk of sexual activity compared to those not pregnant/postpartum, which slowly rose to levels closer to not pregnant/postpartum women by the sixth month of the postpartum period.

**Figure 2:**
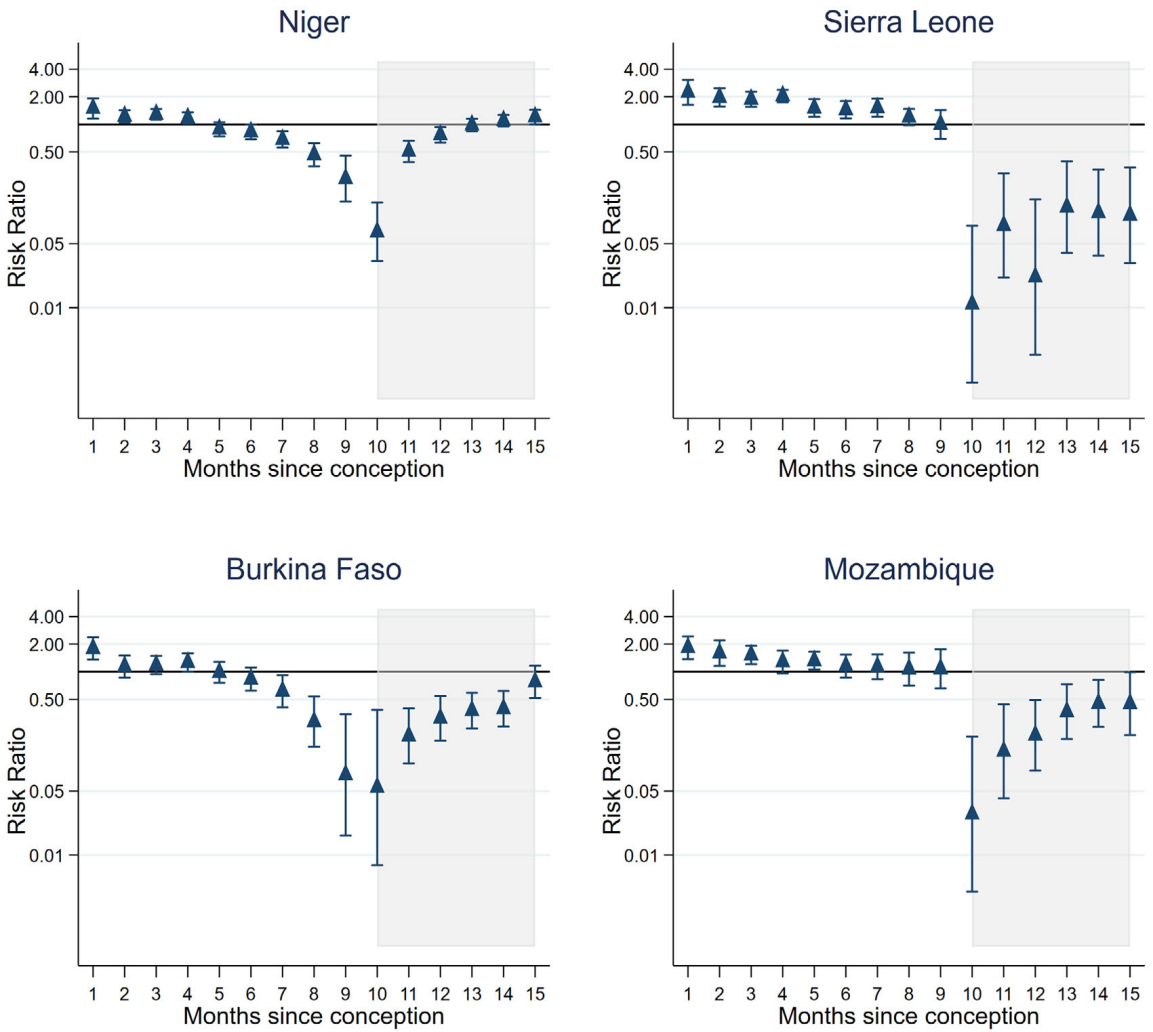
Typical patterns of risk ratios of sexual activity in the last week during the pregnant/postpartum period compared to not pregnant/postpartum, adjusted for 5‐year age group and year of survey in sub‐Saharan Africa. The shaded area is the postpartum period.

**Figure 3:**
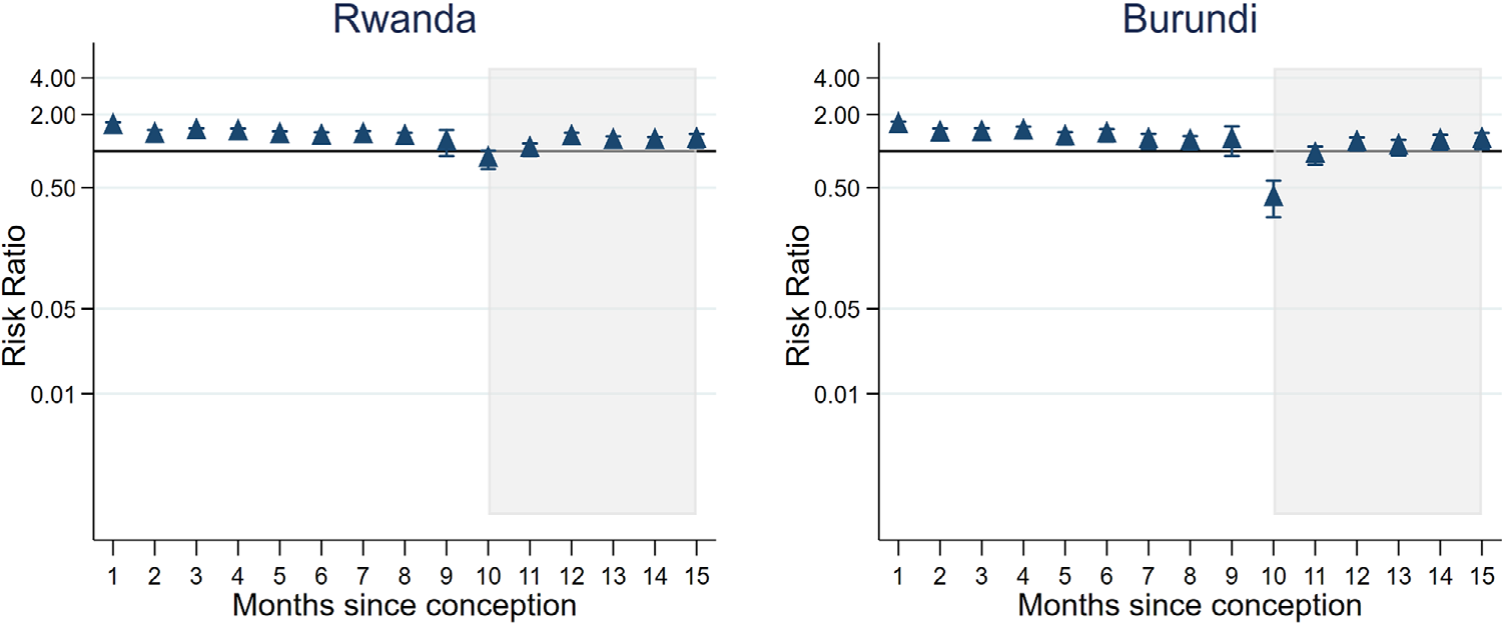
Two atypical patterns of risk ratios of sexual activity in the last week, during the pregnant/postpartum period compared to not pregnant/postpartum, adjusted for 5‐year age group and year of survey, in sub‐Saharan Africa (for Rwanda and Burundi). The shaded area is the postpartum period.

When looking at each country separately, adjusting for 5‐year age group, a number of different patterns emerge (Appendix S1). Figure 2 shows four selected countries giving an example of the main patterns. Niger shows a slow decline in sexual activity of pregnant women compared to those not pregnant/postpartum with lower sexual activity in the second trimester and a rapid drop off in the last month with the risk of sexual activity 0.25 (95% CI = 0.14–0.45) times that of not pregnant/postpartum women. After birth, sexual activity falls further to 0.06 (95% CI = 0.03–0.13) times, then steadily increases over the 6 months postpartum to levels comparable to those not pregnant/postpartum. Burkina Faso shows a similar picture, however, levels of sexual activity in the postpartum period remain lower for the 6 months. In Sierra Leone, there is an increase in sexual activity up to the seventh month of pregnancy, but this is followed by a large drop off in the first month postpartum (aRR = 0.027, 95% CI = 0.007–0.113) and remains much lower than women not pregnant/postpartum throughout the 6 months postpartum. Mozambique follows a similar pattern to Sierra Leone, except levels of sexual activity during the postpartum period rise more quickly over the 6 months, becoming closer to women not pregnant/postpartum. Almost all other countries follow one of these four patterns with differing sizes of risk ratios. Rwanda and Burundi are atypical (Figure 3), with very little difference in sexual activity over the course of the pregnancy/postpartum period. In Rwanda, even in the first month postpartum, the risk of having sex in the last week is only 0.86 (95% CI = 0.72–1.01) times that of not pregnant/postpartum women.

### 3.3 Risk of HIV acquisition per‐condomless‐coital‐act

**Figure 4:**
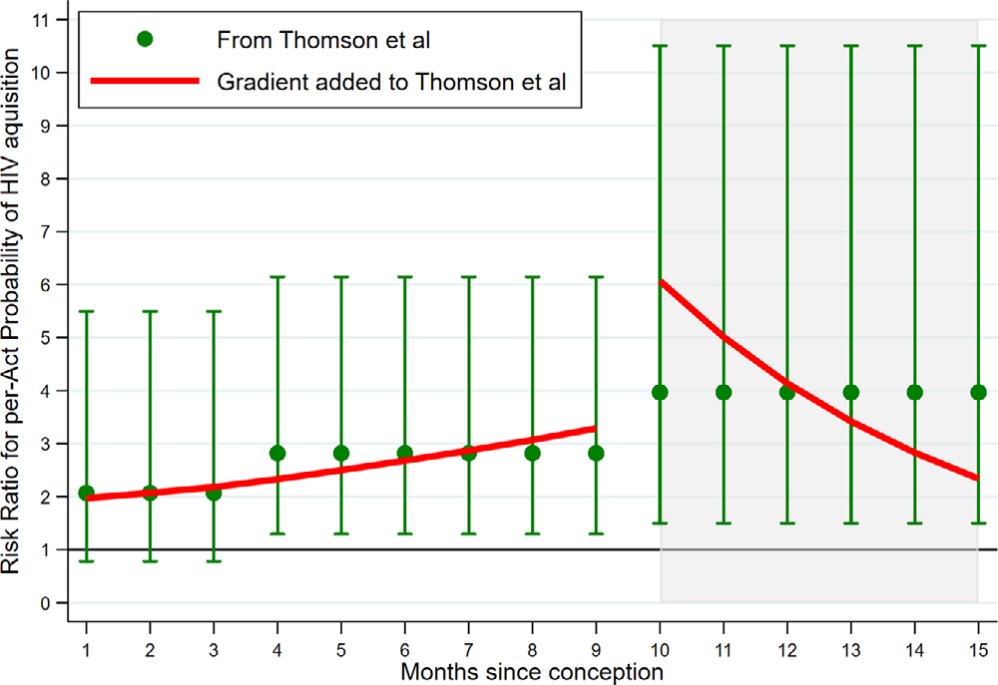
Estimated monthly risk ratios for HIV acquisition per‐condomless‐coital‐act from Thomson et al. [1].

We used a geometric progression between the midpoints of the risk ratio estimates per‐condomless‐coital‐act from Thomson et al. [1]. Figure 4 shows the gradients used as well as keeping the risk ratios constant over each period.

### 3.4 Estimation of population‐level HIV acquisition risk ratios

**Figure 5:**
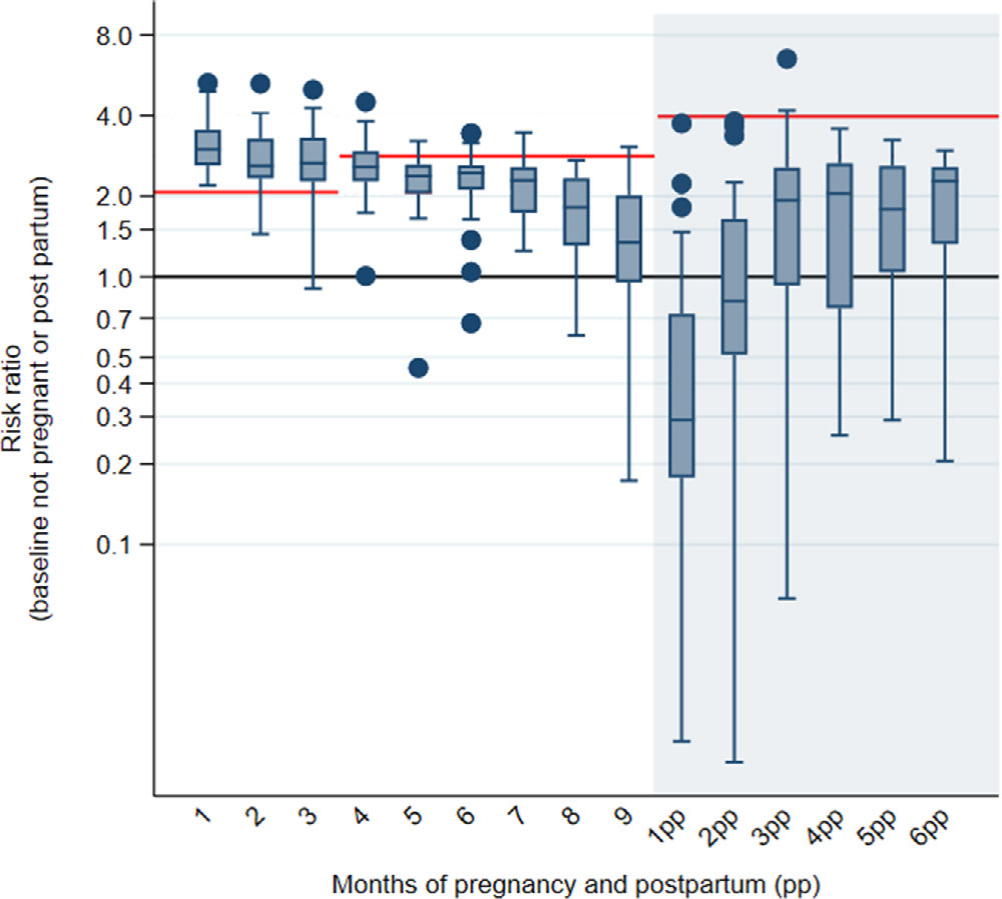
The variation in estimated monthly population risk of HIV acquisition in pregnancy/postpartum compared with not pregnant/postpartum, using per‐condomless‐coital‐act risk probabilities from Thomson et al. and taking into account differences in sexual activity and serodiscordance between the groups, adjusted for 5‐year age group. The red line represents the point estimates for per‐condomless‐coital‐act risk ratio at each stage of pregnancy/postpartum compared to not pregnant/postpartum from Thomson et al [1].

**Figure 6:**
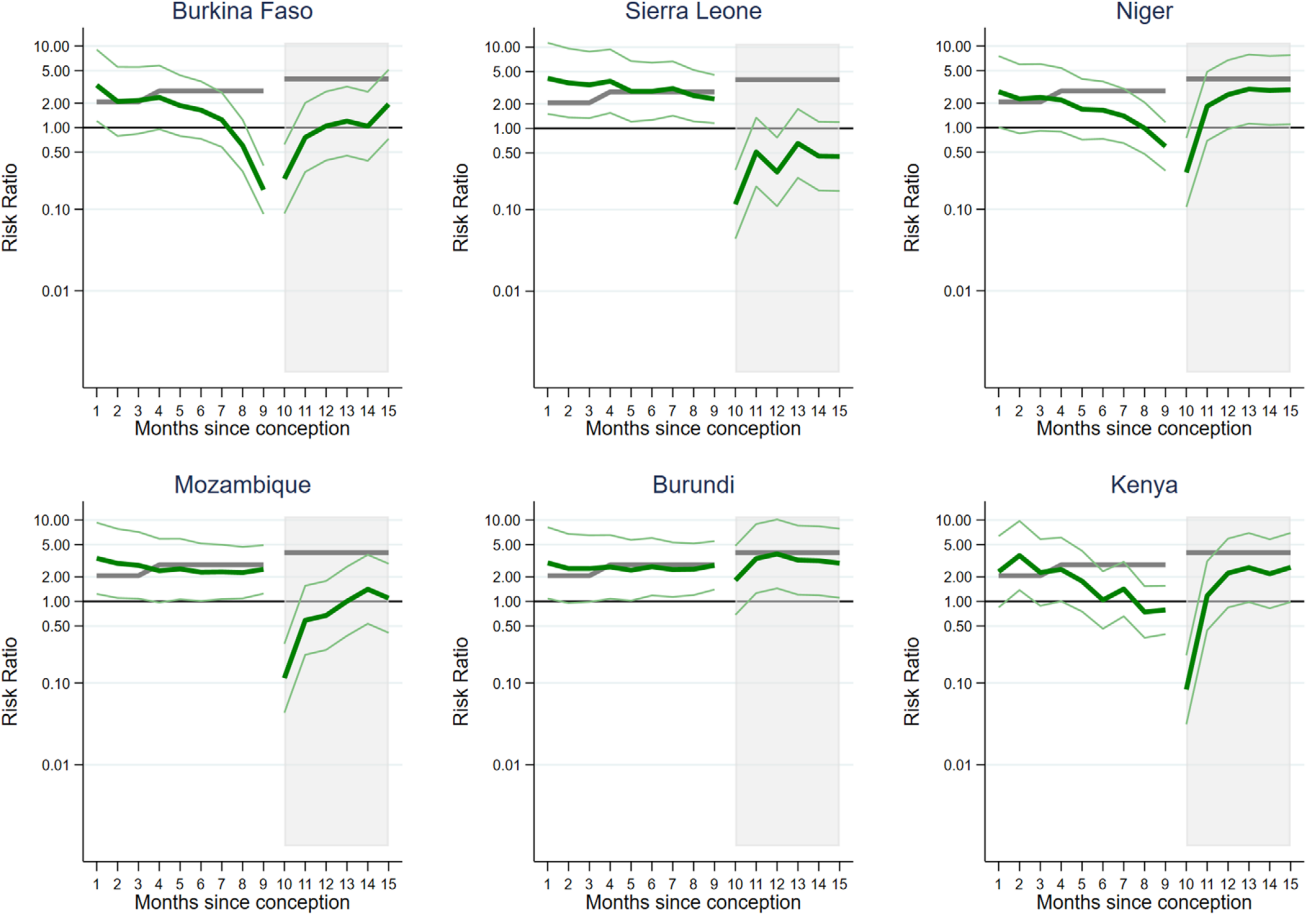
Population‐level risk ratios of HIV acquisition, comparing stages of pregnancy/postpartum to not pregnant/postpartum women, for selected countries. Point estimates using risk ratios per‐condomless‐coital‐act using gradients of risk ratios per‐condomless‐coital‐act and lighter lines the 95% confidence intervals in green. The grey line represents the risk ratio per‐condomless‐coital‐act from Thomson et al [1]. Also, the grey shading is for the months postpartum.

There is a large variation in the modelled population‐level risk ratios comparing stages of the pregnant/postpartum period to women not pregnant/postpartum by country, due to the large variation in levels of sexual activity for each stage (Figure 5, Appendix S1 for individual country patterns). In the first trimester, in all countries, the risk of HIV acquisition is higher than those not pregnant/postpartum due to the increased sexual activity reported at this time. The second trimester patterns diverge; although in general, the risk remains higher than in women not pregnant/postpartum, the risk ratio becomes closer to one. In the third trimester, the relative risk decreases again and, in some countries, reverses with a lower risk of HIV acquisition compared to the not pregnant/postpartum period. This reversal continues in the early postpartum period, where despite the highest risk of per‐condomless‐coital‐act HIV acquisition from Thomson et al., there is a much lower risk of HIV acquisition, compared to not pregnant/postpartum women. Although the overall pattern remains the same over most countries, there are large differences in the risk ratios and timing of lowered HIV acquisition risk (Figures 6, Appendix S1).

### 3.5 Estimation of population‐level acute infection ratios

**Figure 7:**
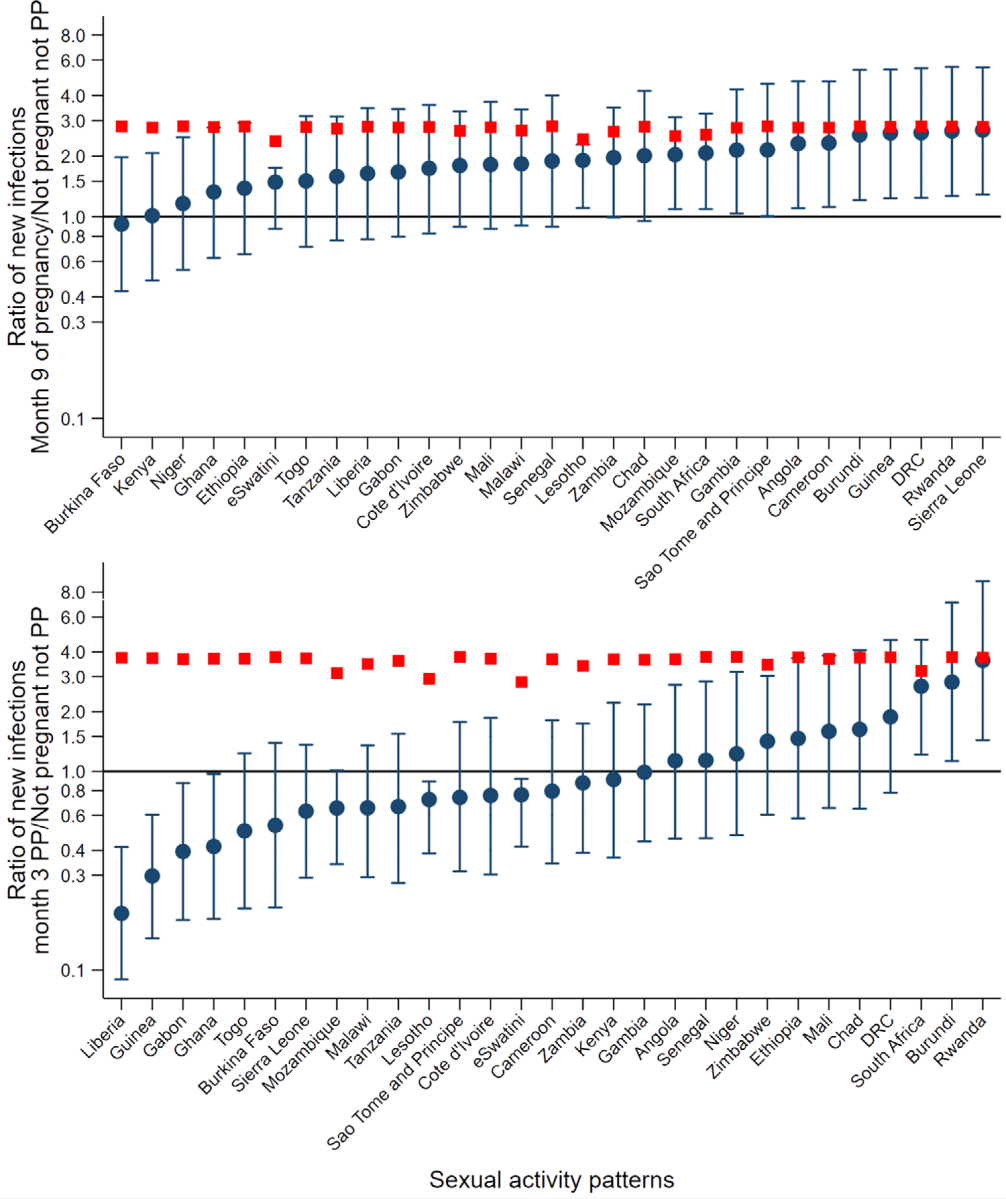
Estimated ratio of acute infections in the ninth month of pregnancy (top) and the third month postpartum (pp) (bottom), compared to not pregnant/postpartum women, assuming no ART initiation. Estimates use per‐condomless‐coital‐act probability taking into account differences in sexual activity in the last week and HIV‐serodiscordancy with a partner, assuming an acute infection window of 3 months. Shown for each country given their sexual activity patterns (blue). Confidence intervals represent uncertainty around the per‐condomless‐coital‐act risk ratio estimates. Also plotted is the point estimate of the per‐condomless‐coital‐act risk ratios from Thomson et al [1] (red).

Estimated population‐level acute infection ratios for the last month of pregnancy and the breastfeeding postpartum period show a slightly different pattern to the population‐level HIV acquisition risk ratio pattern, as they are affected by the HIV acquisition ratios in the 3 months prior to each month's ratio. Overall risk ratios were still considerably lower than that would be found using per‐condomless‐coital‐act risk ratios for most countries at most stages where there is a risk of vertical transmission (Appendix S1). The estimates show, in general, that in the last month of pregnancy there are a greater number of acute infections compared to what would be expected in women not pregnant/postpartum (Figure 7a), but for many countries this switches to fewer infections in the postpartum period (Figure 7b shows the third month, see Appendix S1 for all months). Both these figures demonstrate the widely varying patterns by country.

## 4 Discussion

This analysis shows that, in most countries in sub‐Saharan Africa, during periods of greatest risk for vertical transmission, population‐level risk of HIV acquisition and the resulting acute infections, in pregnant/postpartum women relative to not pregnant/postpartum will be lower than would be assumed solely from per‐condomless‐coital‐act relative risk estimates. Without taking into account sexual activity and serodiscordancy, estimates and projections of the paediatric HIV epidemic using risks from per‐condomless coital‐act studies directly would, in most countries, overestimate vertical transmission, with potential impact on PMTCT policies and interventions.

There is a great variability across countries in sub‐Saharan Africa in sexual activity patterns during pregnancy and the postpartum period, largely due to differences in postpartum abstinence, but differences are also seen towards the end of pregnancy. This gives strong evidence that at a population level there will be wide variation in relative risk of HIV acquisition in pregnancy and postpartum compared to those not pregnant/postpartum. It is also likely to yield regional differences within countries, where postpartum abstinence practices vary.

We found that within couples living together, HIV‐negative pregnant and early postpartum women were less likely to have an HIV‐positive partner, with an even stronger reduction toward the end of pregnancy, though there was not enough power to detect a difference between pregnancy periods. Differences in the risk of having an HIV‐positive partner in this group are most likely due to the increased exposure to HIV infection by a positive partner prior to and at the beginning of pregnancy required to become pregnant. Therefore, HIV‐negative women with a positive partner may be more likely to become positive in the lead up to or at the beginning of pregnancy, reducing the number of HIV‐negative women with a positive partner during pregnancy and postpartum. The difference in the risk of having an HIV‐positive partner moved closer to the null over the postpartum period, possibly indicating that new infections are occurring in the partners of HIV‐negative women at this time. A study in Benin found an increase in the number of condomless extra‐marital partnerships with an increase in postpartum abstinence [18] and in in‐depth interviews in Malawi, women discuss the conflict between traditional abstinence in pregnancy and the postpartum period and the risk their partner may seek sexual intercourse outside the partnership in their absence [19].

The population‐level estimates presented here are likely to be upper estimates as there are many factors not accounted for. There is evidence that risk behaviour associated with HIV acquisition is reduced in women who are pregnant or postpartum compared to those not pregnant/postpartum [2]. Reduced risk behaviour would further reduce the risk ratio of HIV acquisition in pregnancy comparing pregnant/postpartum women with those not pregnant/postpartum. We were only able to look at the differences in the risk of HIV‐negative women having an HIV‐positive partner by months since conception in cohabiting couples. Non‐cohabiting couples likely have more pronounced differences in the risk of serodiscordancy by months since conception since non‐pregnant women are less likely to be concordant with a positive partner due to lower exposure, either due to an earlier stage of a relationship or less regular sex than those cohabiting. Those who are pregnant with non‐cohabiting partners likely have higher sexual activity and are most likely to be concordant with their partners.

This analysis takes independent probabilities of risk to give final estimated risk ratios, assuming no interdependence, which may not be the case. For example, with increased HIV testing, couples may be more aware of their status and, therefore, protect themselves more if there is discordancy, and this may be more likely in couples where the woman is pregnant or postpartum due to increased interaction with services at this time. It also assumes that HIV‐positive partners of pregnant postpartum women have the same distribution of ART use and viral load as not pregnant/postpartum women. If positive partners of HIV‐negative women who are pregnant or postpartum are more likely to be on ART, the risk of HIV acquisition relative to those not pregnant/postpartum will be lower than estimated. If the postpartum period increases the risk of HIV acquisition in the partner and resumption of sexual intercourse begins before the end of breastfeeding, this could increase the risk of HIV acquisition in postpartum women, due to both the per‐condomless‐coital‐act risk of transmission and the acute infection stage of the partner. However, if postpartum abstinence is associated with increased risk of a newly infected HIV‐positive partner and women do not resume sexual relations until after breastfeeding, it will remain protective in terms of the risk of vertical transmission and cause an overestimation in the risk ratios, as this would make those abstaining more likely to be the ones with the HIV‐positive partner.

We provide population‐level estimates of ratios of acute infections assuming no women with incident infection initiated ART, which will overestimate the number of acute infections, particularly in the last month of pregnancy. Many women coming to ANC will have learned their HIV status and have been put on ART, therefore, there is no longer an acute infection by the time there is most risk of vertical transmission.

This analysis demonstrates how sexual activity patterns and differences in discordancy between pregnant and postpartum women and those not pregnant/postpartum can explain some of the differences found between per‐condomless‐coital‐act studies and general population studies. It helps explain the large heterogeneity found across all studies in the systematic review by Graybill et al [6]. It also shows the complexity of defining a population level increased risk of HIV acquisition in pregnancy, given that consideration of just two factors alone can make much of the postpartum period protective for many of the countries considered and reduces considerably the risk ratio found in the per‐condomless‐coital‐act sero‐discordant couples study by Thomson et al. [1].

## 5 Conclusions

Currently Spectrum, the model used by UNAIDS for estimating and projecting the HIV epidemic [20] assumes that the number of acute infections in pregnant and breastfeeding women is the same as in not pregnant/postpartum women for vertical transmission estimates. This analysis shows that ratios directly taken from per‐condomless‐coital‐act studies, to estimate acute infection ratios, should not be adopted, but it also shows that it is likely that in many regions a ratio of one will not be correct and that it will vary over the pregnant/breastfeeding period. However, we are not able to recommend a replacement parameter due to the complexity and variation of sexual behaviour, differences in HIV testing policies and breastfeeding practices and how all these factors interact with each other and change over time. More research is needed to consider how to incorporate it into spectrum.

We have shown that population‐level risks of HIV acquisition in pregnancy and postpartum are lower than those found in per‐coital‐condomless‐act estimates when compared to those not pregnant/postpartum at the time when there is the greatest risk of vertical transmission and that this relationship varies considerably in different settings and at different times during the pregnancy.

## COMPETING INTERESTS

The authors declare no conflict of interests.

## AUTHORS’ CONTRIBUTIONS

MM & MIM conceived the analysis, MMar designed the analysis, KR commented on analysis design, MM analysed the data, MM, KR and MIM wrote the article.

## Supporting information

**Appendix S1**. Additional information for sections of the methods
