# Supplementary material for: HIV acquisition in pregnancy: implications for mother‐to‐child transmission at the population level in sub‐Saharan Africa
Source: J Int AIDS Soc. 2021 Sep 21;24(Suppl 5):e25783. doi: 10.1002/jia2.25783 (PMC8454677; doi:10.1002/jia2.25783)
Supplement: Supplementary file 1 — Appendix S1. Additional information for sections of the methods [file JIA2-24-e25783-s001.pdf]

SUPPORTING INFORMATION

HIV Acquisition in Pregnancy – implications for mother to child transmission at the population level in sub-Saharan Africa

Milly Marston[1§], Kathryn Risher[1], Mary Mahy[2]

1 London School of Hygiene and Tropical Medicine, London
2 UNAIDS, Geneva

Additional information for sections of the methods

Data

We used data from 60 Demographic and Health Surveys (DHS) and AIDS indicator surveys (AIS) conducted in 32 sub-Saharan African countries between 2000 and 2017 in which HIV testing outcomes were available. DHS and AIS are nationally representative household surveys. All analyses account for the two-stage cluster sampling survey design and use the HIV weights or male sample weights, as appropriate, provided by DHS. In pooled analysis, surveys are re-weighted so that each survey contributes equally toward the analysis. From these surveys, 53 surveys from 29 countries were available with both data linking couples and HIV status data. The couple data are linked data from the individual men and women files where they have declared to be married (living together) at the time of the survey. A respondent may have a regular partner, or a spouse who resides de jure in the same household, but if, at the time of the survey that person is not a de facto cohabiting partner then they are not included as there is little information about the partner. Women included in the analysis were aged 15-49.

Analysis

Definitions of pregnancy and the postpartum period

The risk of mother to child transmission occurs in the period of pregnancy and during breastfeeding in the postpartum period. Women were classified as pregnant if they answered yes to "are you currently pregnant", duration of the current pregnancy was also asked in months. Postpartum women were identified by looking at the date of birth of the last child and date of interview. We randomly impute the day of birth of the child across the month they were born in. If the month they were born is the same as the month of interview the day of birth was randomly imputed to be between the first of the month and the day of interview. Months postpartum were calculated from this (assuming a month is 30 days), as long as the woman was not currently pregnant. Using this information, we created a maternity status variable "months since conception" with months 1-9 being pregnancy and months 10-15 the six months postpartum. We further refined the postpartum period to include only women who reported they were currently breastfeeding. For some analyses this variable was grouped into three-month periods, the three trimesters of pregnancy and early and late postpartum.

Analysis of differences in couple serodiscordancy by months since conception

We repeated the analysis restricting to HIV negative women who reported having sex in the last week and no condom use in order to look at any variation in the levels of discordancy in HIV negative women by sexual activity.

Table S1: Summary statistics used as inputs in scenarios by country

Country	Median postpartum abstinence (months)[a]	Estimated 15-49 year old female HIV incidence in 2019 per 1000 (Uncertainty bounds)[b]
Angola	4.1	1.99(1.37-2.68)
Burkina Faso	7.7	0.20(0.13-0.30)
Burundi	1.2	0.23(0.14-0.37)
Cameroon	5.8	1.44(1.15-1.80)
Chad	2.3	0.59(0.37-0.92)
Cote d'Ivoire	6.1	1.02(0.67-1.49)
Democratic Republic of Congo	3.7	0.48(0.29-0.74)
Ethiopia	2.7	0.29(0.16-0.52)
eSwatini	4.6	13.97(10.85-18.01)
Gabon	5.8	1.54(0.71-2.77)
Gambia	6.0	1.99(1.29-3.06)
Ghana	5.9	1.42(0.69-2.59)
Guinea	21.1	0.85(0.59-1.19)
Kenya	3.1	1.84(1.17-2.86)
Lesotho	7.2	13.86(11.64-16.65)
Liberia	11.6	0.84(0.54-1.28)
Malawi	4.6	4.23(3.49-4.82)
Mali	2.2	1.50[c]
Mozambique	9.8	10.53(6.38-16.78)
Namibia	6.3	6.64(5.49-7.87)
Niger	2.2	0.08(0.06-0.12)
Rwanda	0.9	0.88(0.63-1.24)
Sao Tome and Principe	2.1	0.14[c]
Senegal	3.6	0.13(0.10-0.18)
Sierra Leone	17.4	1.06(0.77-1.40)
South Africa	4.7	9.63(8.69-10.39)
Togo	5.4	1.08(0.74-1.61)
United Republic of Tanzania	3.8	3.07(2.71-3.47)
Zambia	3.0	7.12(6.02-8.70)
Zimbabwe	2.6	5.64(3.89-7.84)

[a]Source, STATcomplier, DHS program https://www.statcompiler.com/en/ accessed 10th December2019
[b]Source: AIDSinfo, UNAIDS https://aidsinfo.unaids.org/ accessed 11th January 2020

[c]Data unavailable for 2020, estimates from 2018 accessed 10th November 2019

Additional Results

Serodiscordancy by months since conception

Overall, the percentage of all HIV negative women with an HIV positive partner in the couples dataset varies from 0.2% (95%CI 0.1-0.4%) in Niger 2012 to 13.0% (95%CI 10.3-16.3%) in Lesotho 2009-10. The proportion of HIV negative women with an HIV positive partner is consistently lower in pregnant women compared to not pregnant or postpartum women over nearly all surveys, although confidence intervals overlap (Table S2). This is true also for many countries for the early and late postpartum period, however the confidence intervals are very wide.

Table S2: Cohabiting couple data from all surveys with proportion of HIV negative women with HIV positive partner by maternity status

Survey Country and year	Unwei N	% of HIV negative women with HIV positive partner by Maternity Status				
		All Women	Not pregnant	Pregnant	Early PP	Late PP
**Southern**						
Angola, 2015-16	2102	0.8 (0.4-1.5)	1.0 (0.5-1.9)	0.4 (0.1-1.1)	0.2 (0.0-1.6)	0.6 (0.1-4.2)
Lesotho, 2004-05	437	11.9 (9.2-15.3)	12.5 (9.3-16.5)	7.2 (2.8-17.4)	14.4 (3.4-44.7)	14.6 (3.1-47.3)
Lesotho, 2009-10	611	13.0 (10.3-16.3)	14.2 (11.2-17.8)	7.4 (2.5-19.8)	2.5 (0.3-16.8)	9.3 (2.7-27.0)
Lesotho, 2014	508	11.1 (8.4-14.6)	12.0 (9.1-15.8)	2.5 (0.3-16.1)	0.0 (.- .)	13.6 (2.4-49.7)
Mozambique, 2015	1989	5.3 (4.2-6.8)	5.6 (4.3-7.3)	4.6 (2.5-8.5)	4.4 (1.7-10.6)	4.0 (1.3-11.4)
Namibia, 2013	897	9.5 (7.0-12.7)	10.2 (7.4-13.9)	4.5 (1.8-10.5)	6.9 (1.6-25.0)	7.3 (2.1-22.6)
South Africa, 2016	236	7.0 (3.5-13.6)	7.1 (3.4-14.2)	0.0 (.- .)	63.2 (12.8-95.2)	0.0 (.- .)
eSwatini, 2006-07	414	12.3 (9.2-16.2)	13.8 (10.2-18.5)	3.0 (0.8-10.7)	27.9 (6.6-67.8)	4.4 (0.6-25.8)
Zambia, 2007	1992	7.5 (6.3-8.9)	7.8 (6.4-9.5)	6.1 (3.9-9.4)	3.2 (1.1-8.8)	11.2 (6.8-17.9)
Zambia, 2013-14	5693	7.0 (6.1-8.0)	7.0 (6.0-8.2)	6.7 (4.9-9.2)	7.4 (4.3-12.5)	7.1 (4.4-11.3)
Zimbabwe, 2005-06	1494	9.6 (7.9-11.7)	10.2 (8.1-12.8)	7.4 (4.6-11.9)	11.0 (5.1-22.2)	5.9 (2.6-13.2)
Zimbabwe, 2010-11	1996	7.8 (6.6-9.1)	8.1 (6.8-9.5)	6.6 (4.0-10.7)	7.1 (3.6-13.7)	7.9 (4.4-13.8)
Zimbabwe, 2015	2510	5.9 (4.9-7.1)	6.5 (5.3-7.8)	3.4 (1.7-6.4)	1.9 (0.6-6.0)	4.9 (2.4-9.9)
**Eastern**						
Burundi, 2010-11	1889	0.4 (0.2-0.9)	0.4 (0.2-1.0)	0.5 (0.1-2.1)	0.0 (.- .)	1.0 (0.1-7.0)
Burundi, 2016-17	3454	0.6 (0.3-1.0)	0.4 (0.2-0.7)	1.2 (0.3-4.6)	1.8 (0.5-6.3)	0.0 (.- .)
Kenya, 2003	997	3.2 (2.1-4.8)	3.2 (2.0-5.2)	3.2 (1.0-9.5)	2.4 (0.6-9.4)	3.7 (0.9-13.8)
Kenya, 2008-09	1143	3.0 (2.0-4.5)	2.7 (1.7-4.1)	2.2 (0.7-7.0)	9.0 (3.1-23.4)	4.5 (1.0-17.7)
Malawi, 2004-05	1140	6.6 (5.0-8.7)	7.0 (5.2-9.5)	6.2 (3.3-11.4)	3.7 (0.9-14.2)	6.1 (1.8-18.9)
Malawi, 2010	3012	5.2 (4.3-6.3)	5.3 (4.3-6.7)	4.6 (2.8-7.4)	3.0 (1.0-8.1)	6.7 (2.8-15.2)
Malawi, 2015-16	2925	4.9 (4.0-6.0)	5.4 (4.3-6.7)	3.3 (1.9-5.7)	3.4 (1.4-8.1)	2.6 (0.9-7.2)
Rwanda, 2005	2079	1.4 (1.0-2.1)	1.6 (1.0-2.5)	0.9 (0.3-2.6)	0.6 (0.1-2.4)	1.6 (0.5-5.1)
Rwanda, 2010-11	2715	1.4 (1.0-1.9)	1.6 (1.1-2.2)	0.6 (0.1-2.4)	1.9 (0.5-7.5)	0.0 (.- .)
Rwanda, 2014-15	2785	1.5 (1.1-2.0)	1.5 (1.1-2.1)	1.4 (0.6-3.2)	0.0 (.- .)	3.0 (1.1-7.7)
**Western and Ethiopia**						
Burkina Faso, 2003	2127	0.9 (0.5-1.6)	0.8 (0.4-1.7)	0.4 (0.1-1.7)	0.0 (.- .)	3.2 (0.8-12.0)
Burkina Faso, 2010	4847	0.5 (0.3-0.8)	0.6 (0.4-0.9)	0.5 (0.1-1.5)	0.4 (0.1-3.1)	0.0 (.- .)
Cameroon, 2004	1907	2.5 (1.9-3.3)	2.8 (2.0-3.9)	1.6 (0.7-3.8)	0.7 (0.1-4.5)	2.7 (0.9-7.6)
Cameroon, 2011	2699	2.9 (2.3-3.7)	3.3 (2.5-4.3)	1.4 (0.6-3.0)	1.0 (0.2-4.2)	4.5 (1.9-10.4)
Chad, 2014-15	2826	0.9 (0.5-1.4)	1.0 (0.6-1.7)	0.6 (0.1-2.4)	0.0 (.- .)	1.2 (0.4-3.7)
Democratic Rep. Congo, 2007	2115	0.6 (0.3-1.0)	0.5 (0.3-1.0)	0.2 (0.0-1.3)	1.1 (0.2-7.8)	1.6 (0.5-5.1)
Democratic Rep. Congo, 2013-14	4265	0.5 (0.3-0.9)	0.4 (0.2-1.0)	1.0 (0.4-2.7)	0.3 (0.0-2.4)	0.4 (0.1-1.6)
Cote d'Ivoire, 2011-12	1724	2.9 (2.0-4.2)	3.1 (2.1-4.7)	2.7 (1.2-6.0)	2.9 (0.4-17.3)	0.8 (0.2-3.4)
Ethiopia, 2005	2441	0.8 (0.5-1.3)	0.9 (0.5-1.6)	0.3 (0.0-1.7)	0.0 (.- .)	1.1 (0.2-7.5)
Ethiopia, 2010-11	6091	0.4 (0.3-0.7)	0.5 (0.3-0.9)	0.1 (0.0-0.4)	0.0 (0.0-0.1)	0.4 (0.1-3.0)

Ethiopia, 2016	5416	0.4 (0.2-0.7)	0.5 (0.3-0.9)	0.0 (.- .)	0.0 (0.0-0.1)	0.0 (0.0-0.1)
Gabon, 2012	1771	2.1 (1.4-3.2)	2.5 (1.7-3.8)	0.6 (0.2-1.7)	0.0 (.- .)	1.6 (0.2-10.6)
Gambia, 2013	1161	1.6 (0.8-3.4)	1.3 (0.6-2.9)	1.7 (0.2-11.5)	1.4 (0.2-9.6)	5.3 (1.3-19.6)
Ghana, 2003	1776	1.5 (1.0-2.2)	1.8 (1.2-2.7)	0.2 (0.0-1.8)	1.1 (0.1-7.5)	0.0 (.- .)
Ghana, 2014	1681	0.8 (0.4-1.6)	0.8 (0.4-1.7)	0.0 (.- .)	3.4 (0.6-18.0)	0.0 (.- .)
Guinea, 2005	1834	0.9 (0.5-1.7)	0.9 (0.4-1.8)	0.6 (0.1-2.5)	0.7 (0.1-4.6)	2.8 (0.9-8.8)
Guinea, 2012	2145	1.0 (0.6-1.8)	1.0 (0.5-1.9)	1.7 (0.7-3.9)	0.0 (.- .)	1.1 (0.3-4.4)
Liberia, 2006-07	2219	0.7 (0.4-1.2)	0.9 (0.6-1.6)	0.0 (.- .)	0.0 (.- .)	0.2 (0.0-1.5)
Liberia, 2013	1632	1.7 (0.9-3.0)	1.8 (0.9-3.6)	1.4 (0.5-3.7)	0.6 (0.1-4.2)	0.5 (0.1-3.5)
Mali, 2006	2440	0.3 (0.2-0.7)	0.4 (0.2-0.8)	0.4 (0.1-2.8)	0.0 (.- .)	0.0 (.- .)
Mali, 2012-13	2565	0.8 (0.5-1.5)	0.5 (0.2-1.1)	2.5 (1.2-5.0)	0.0 (.- .)	1.9 (0.4-8.3)
Niger, 2006	2019	0.6 (0.3-1.1)	0.6 (0.3-1.3)	0.3 (0.1-1.2)	0.8 (0.2-3.5)	0.7 (0.2-2.7)
Niger, 2012	2500	0.2 (0.1-0.4)	0.2 (0.1-0.5)	0.3 (0.0-2.2)	0.0 (.- .)	0.0 (.- .)
Sao Tome and Principe, 2008-09	869	2.0 (1.1-3.6)	2.5 (1.4-4.5)	0.9 (0.1-6.2)	0.0 (.- .)	0.0 (.- .)
Senegal, 2005	1190	0.3 (0.1-0.6)	0.3 (0.1-0.7)	0.0 (.- .)	0.0 (.- .)	0.9 (0.1-6.1)
Senegal, 2010-11	1570	0.6 (0.2-1.4)	0.7 (0.3-1.9)	0.0 (0.0-0.3)	0.3 (0.0-2.3)	0.6 (0.1-4.2)
Senegal, 2017	2304	0.4 (0.2-0.9)	0.5 (0.2-1.2)	0.0 (.- .)	0.0 (.- .)	0.5 (0.1-3.4)
Sierra Leone, 2008	1556	0.7 (0.3-1.8)	0.9 (0.4-2.4)	0.0 (.- .)	0.0 (.- .)	0.0 (.- .)
Sierra Leone, 2013	3391	1.3 (0.9-2.1)	1.4 (0.9-2.4)	1.3 (0.5-3.4)	0.7 (0.1-4.7)	1.0 (0.2-3.9)
Togo, 2013-14	2168	1.1 (0.7-1.6)	1.1 (0.7-1.8)	0.9 (0.2-3.9)	1.8 (0.4-7.3)	0.0 (.- .)

Table S3: Risk ratio for having an HIV positive partner for HIV negative women who reported sexual intercourse in the last week and do not report condom use.

	Crude RR	(95%CI)	Adjusted* RR	(95%CI)
Maternity Status				
Not pregnant/postpartum	1		1	
First Trimester	0.89	(0.68-1.16)	1.03	(0.80-1.35)
Second Trimester	0.64	(0.50-0.83)	0.74	(0.57-0.95)
Third Trimester	0.62	(0.45-0.87)	0.68	(0.49-0.95)
Early Postpartum	0.54	(0.32-0.89)	0.80	(0.49-1.31)
Late Postpartum	0.65	(0.49-0.88)	0.80	(0.60-1.07)
Maternity status broad grouping				
Not pregnant/postpartum	1		1	
Early Pregnancy	0.88	(0.68-1.16)	1.03	(0.80-1.35)
Late Pregnancy	0.63	(0.52-0.78)	0.72	(0.50-0.88)
Postpartum	0.62	(0.48-0.80)	0.80	(0.62-1.03)

*Adjusted for five-year age group, calendar year and country

Table S4: Proportion of all women aged 15-49, currently living with partner (Eligible for DHS couple linking dataset) by maternity status

Region	Survey Country and year	Proportion living with partner			
		Not	Pregnant	Early postpartum	Late postpartum
**Southern**					
	Angola, 2015-16	0.43	0.66	0.64	0.71
	eSwatini, 2006-07	0.24	0.39	0.32	0.35
	Lesotho, 2004-05	0.26	0.51	0.41	0.27
	Lesotho, 2009-10	0.27	0.50	0.38	0.47
	Lesotho, 2014	0.28	0.48	0.31	0.41
	Mozambique, 2015	0.52	0.68	0.65	0.64
	Namibia, 2013	0.28	0.40	0.39	0.29
	South Africa, 2016	0.27	0.33	0.39	0.33
	Zambia, 2007	0.51	0.79	0.71	0.73
	Zambia, 2013-14	0.52	0.76	0.70	0.76
	Zimbabwe, 2005-06	0.39	0.71	0.62	0.61
	Zimbabwe, 2010-11	0.43	0.73	0.69	0.62
	Zimbabwe, 2015	0.44	0.74	0.62	0.72
**Eastern**					
	Burundi, 2010-11	0.45	0.83	0.83	0.82
	Burundi, 2016-17	0.43	0.83	0.82	0.81
	Kenya, 2003	0.43	0.71	0.70	0.62
	Kenya, 2008-09	0.42	0.74	0.52	0.62
	Malawi, 2004-05	0.59	0.84	0.82	0.81
	Malawi, 2010	0.53	0.78	0.72	0.70
	Malawi, 2015-16	0.52	0.77	0.77	0.69
	Rwanda, 2005	0.35	0.84	0.82	0.80
	Rwanda, 2010-11	0.39	0.78	0.75	0.72
	Rwanda, 2014-15	0.42	0.82	0.80	0.69
	Tanzania, 2007-08	0.52	0.82	0.78	0.81
	Tanzania, 2011-12	0.52	0.79	0.72	0.75
**Western and Ethiopia**					
	Burkina Faso, 2003	0.63	0.90	0.87	0.93
	Burkina Faso, 2010	0.66	0.93	0.89	0.88
	Cameroon, 2004	0.47	0.74	0.65	0.72
	Cameroon, 2011	0.44	0.76	0.67	0.69
	Chad, 2014-15	0.57	0.81	0.80	0.82
	Congo Democratic Rep., 2007	0.49	0.82	0.77	0.80
	Congo Democratic Rep., 2013-14	0.46	0.75	0.77	0.69
	Cote d'Ivoire, 2011-12	0.50	0.72	0.59	0.74
	Ethiopia, 2005	0.53	0.92	0.91	0.88
	Ethiopia, 2010-11	0.52	0.90	0.86	0.87
	Ethiopia, 2016	0.55	0.90	0.91	0.86
	Gabon, 2012	0.40	0.54	0.55	0.51
	Gambia, 2013	0.40	0.75	0.64	0.63
	Ghana, 2003	0.41	0.65	0.69	0.63
	Ghana, 2014	0.42	0.66	0.65	0.63
	Guinea, 2005	0.63	0.84	0.82	0.80
	Guinea, 2012	0.57	0.85	0.77	0.71
	Liberia, 2006-07	0.51	0.66	0.64	0.70
	Liberia, 2013	0.46	0.66	0.59	0.61
	Mali, 2006	0.64	0.86	0.80	0.82
	Mali, 2012-13	0.72	0.86	0.87	0.88
	Niger, 2006	0.64	0.79	0.74	0.82
	Niger, 2012	0.67	0.80	0.69	0.78
	Sao Tome and Principe, 2008-09	0.45	0.66	0.72	0.71
	Senegal, 2005	0.37	0.64	0.66	0.60
	Senegal, 2010-11	0.38	0.69	0.64	0.61
	Senegal, 2017	0.35	0.60	0.53	0.62
	Sierra Leone, 2008	0.62	0.75	0.75	0.76
	Sierra Leone, 2013	0.52	0.72	0.65	0.76
	Togo, 2013-14	0.49	0.75	0.74	0.76

Figure S1: Risk of reporting having condomless sex in the last week by months since conception compared to those not pregnant/postpartum for HIV negative women by country adjusted for five-year age group and survey year.

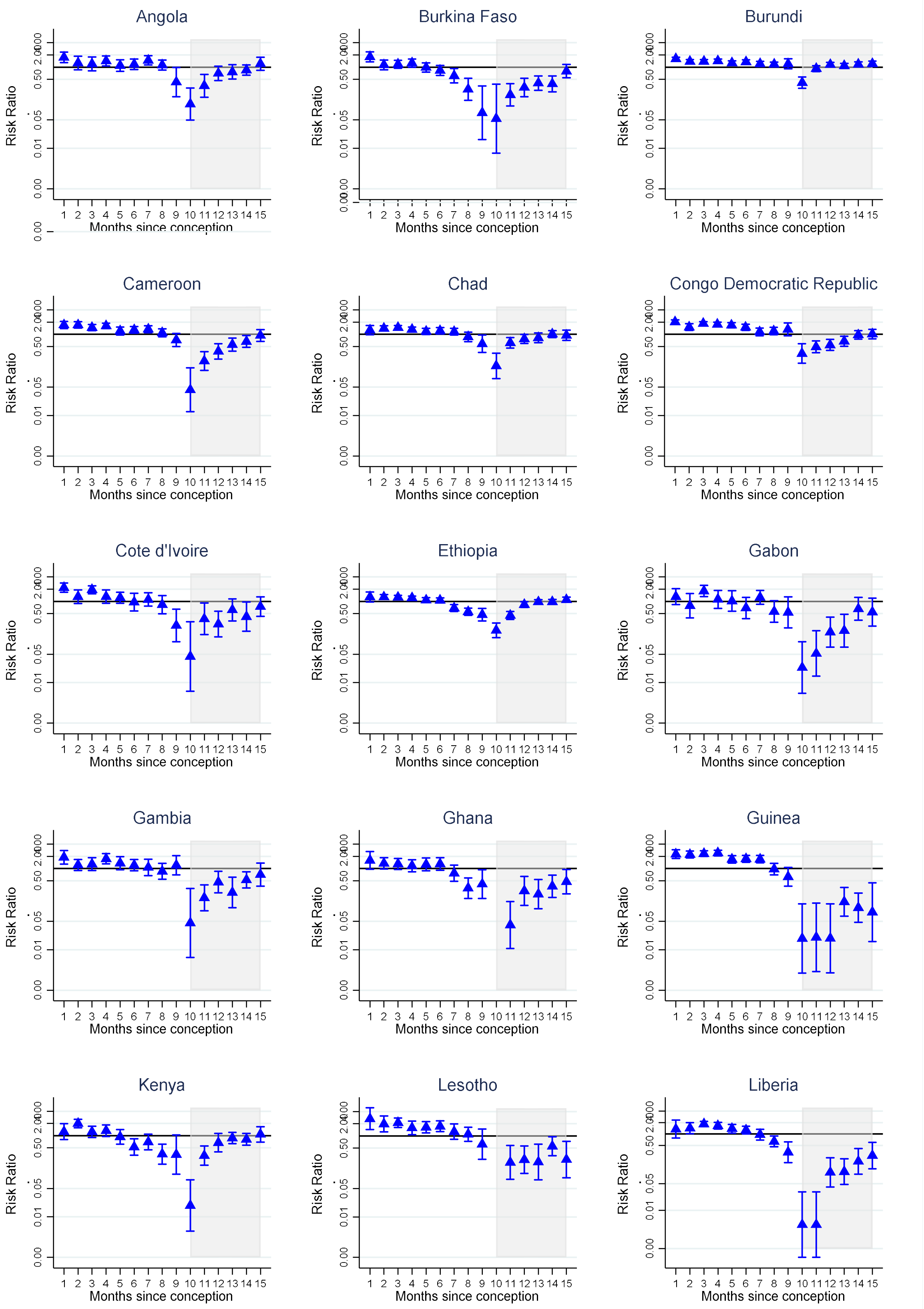

Figure S1 continued: Risk of reporting having condomless sex in the last week by months since conception compared to those not pregnant/postpartum for HIV negative women by country adjusted for five-year age group and survey year.

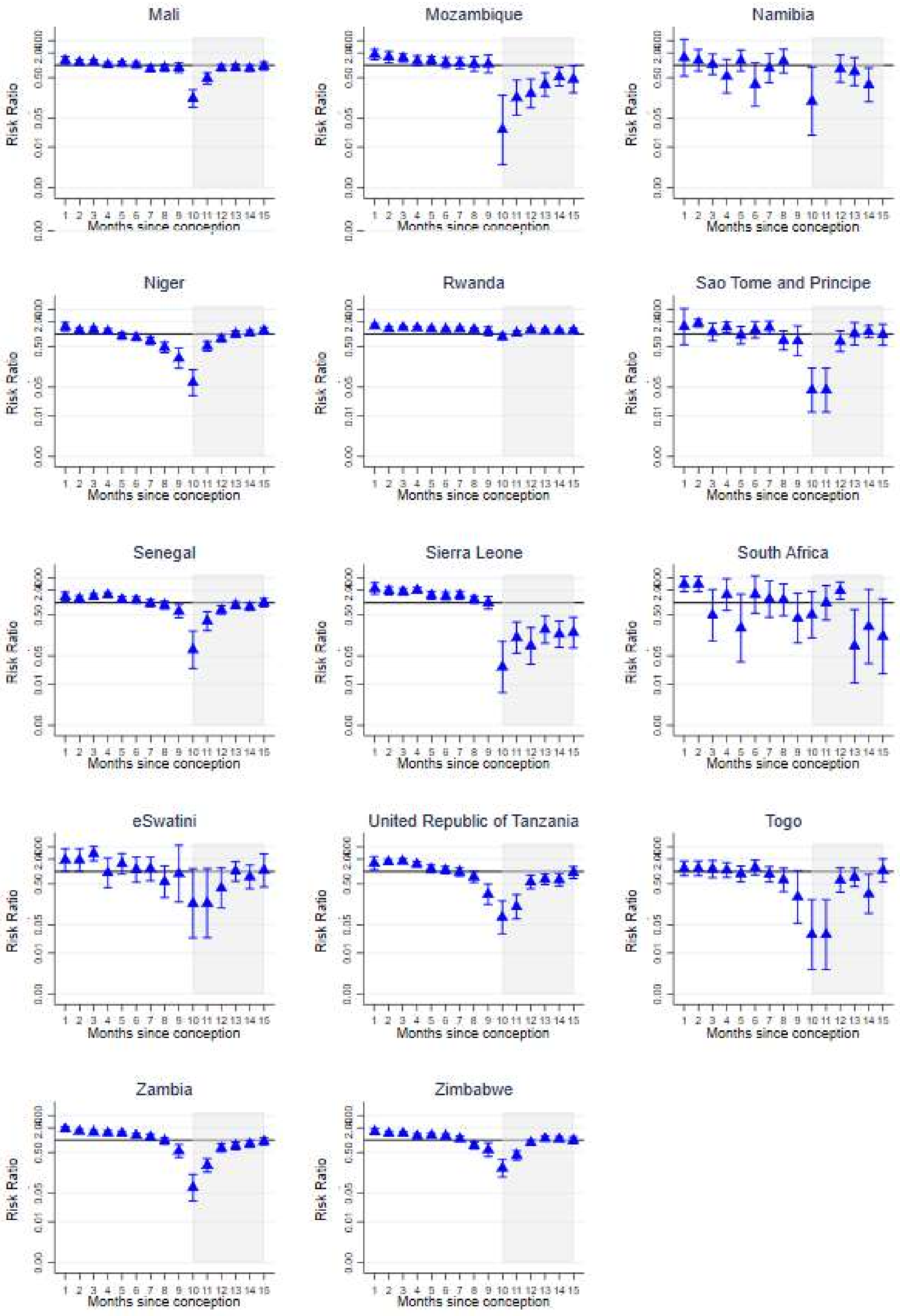

Figure S2: Population level risk ratios adjusted for five-year age group, comparing stages of pregnancy and the postpartum period (shaded in grey) to not pregnant or postpartum women, for selected countries. Thick lines represent point estimates, lighter lines the 95% confidence intervals. Green lines represent estimates using risk ratios per coital act as constant over early pregnancy, late pregnancy and postpartum; red lines represent coital frequency using gradients of risk ratios per coital act. Grey line represents the risk ratio per coital act from Thomson et al. (Note: y-axis on the log scale)

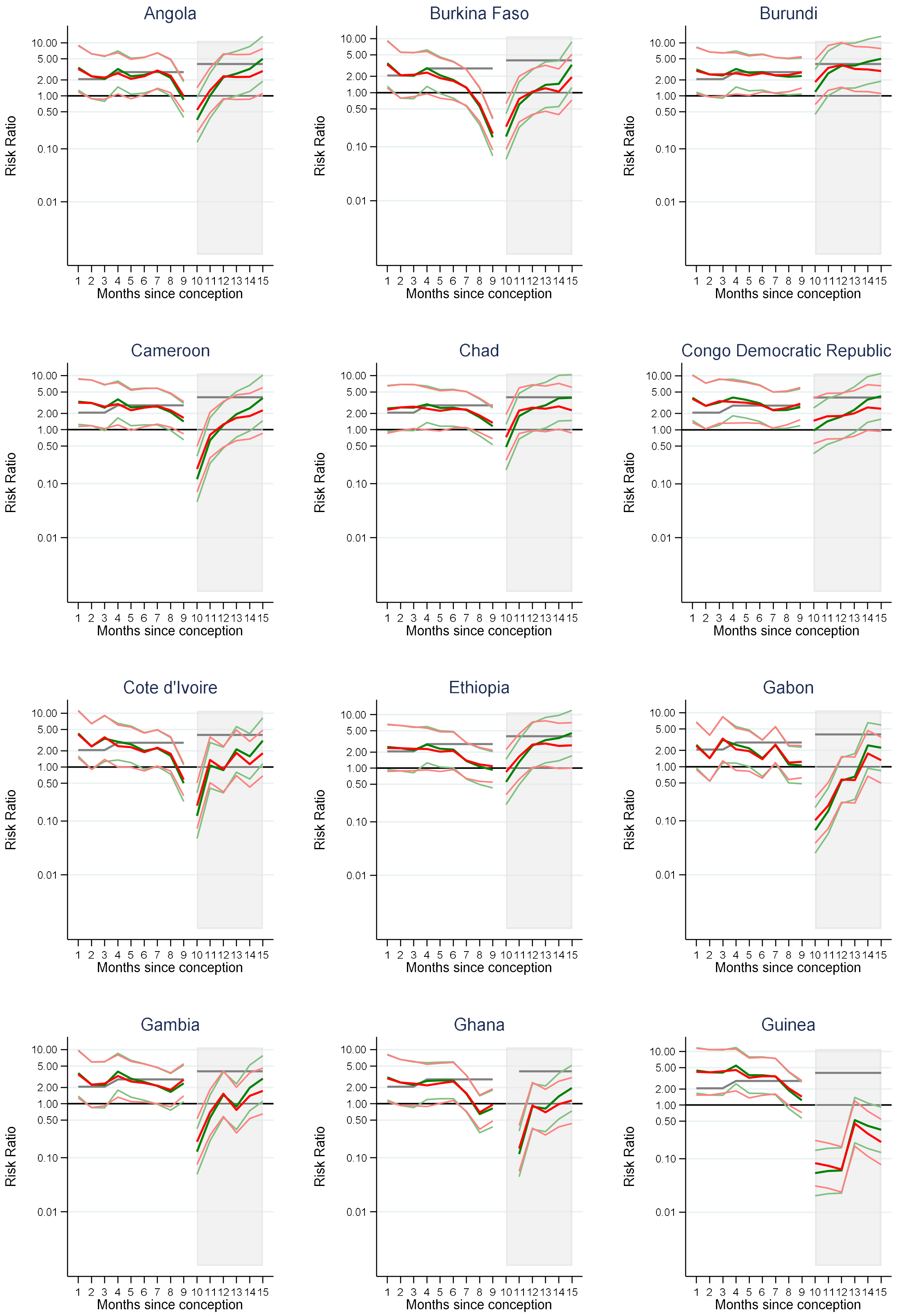

Figure S2 continued: Population level risk ratios for HIV acquisition, comparing stages of pregnancy and the postpartum period (shaded in grey) to not pregnant not postpartum women, for selected countries. Thick lines represent point estimates lighter lines the 95% confidence intervals. Green lines, using risk ratios per coital act as constant over early pregnancy, late pregnancy and postpartum; red lines represent coital frequency using gradients of risk ratios per coital act. Grey line represents the risk ratio per coital act from Thomson et al. (Note: y-axis on the log scale)

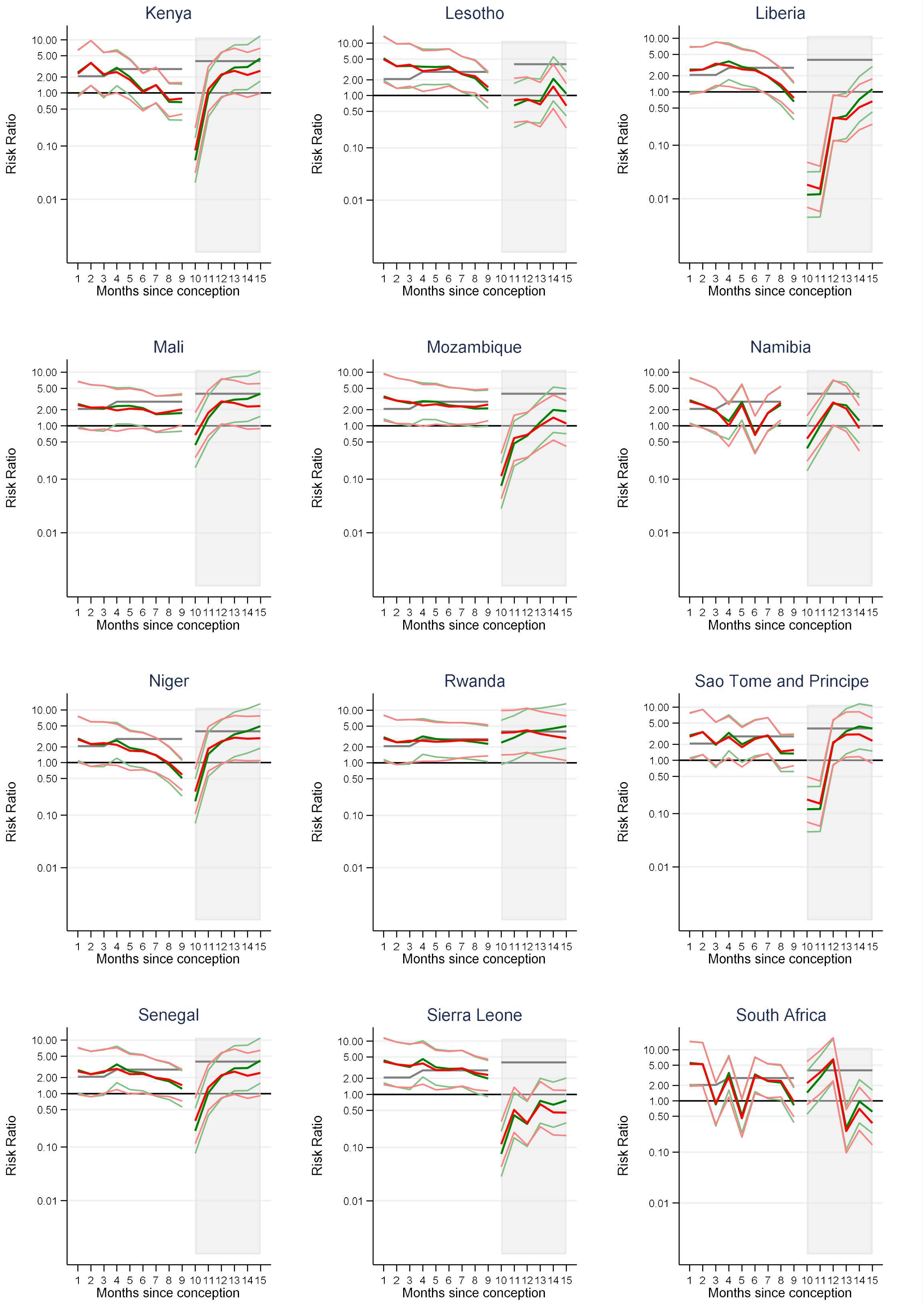

Figure S2 continued: Population level risk ratios for HIV acquisition, comparing stages of pregnancy and the postpartum period (shaded in grey) to not pregnant not postpartum women, for selected countries. Thick lines represent point estimates lighter lines the 95% confidence intervals. Green using risk ratios per coital act as constant over early pregnancy, late pregnancy and postpartum; red lines represent coital frequency using gradients of risk ratios per coital act. Grey line represents the risk ratio per coital act from Thomson et al. (Note: y-axis on the log scale)

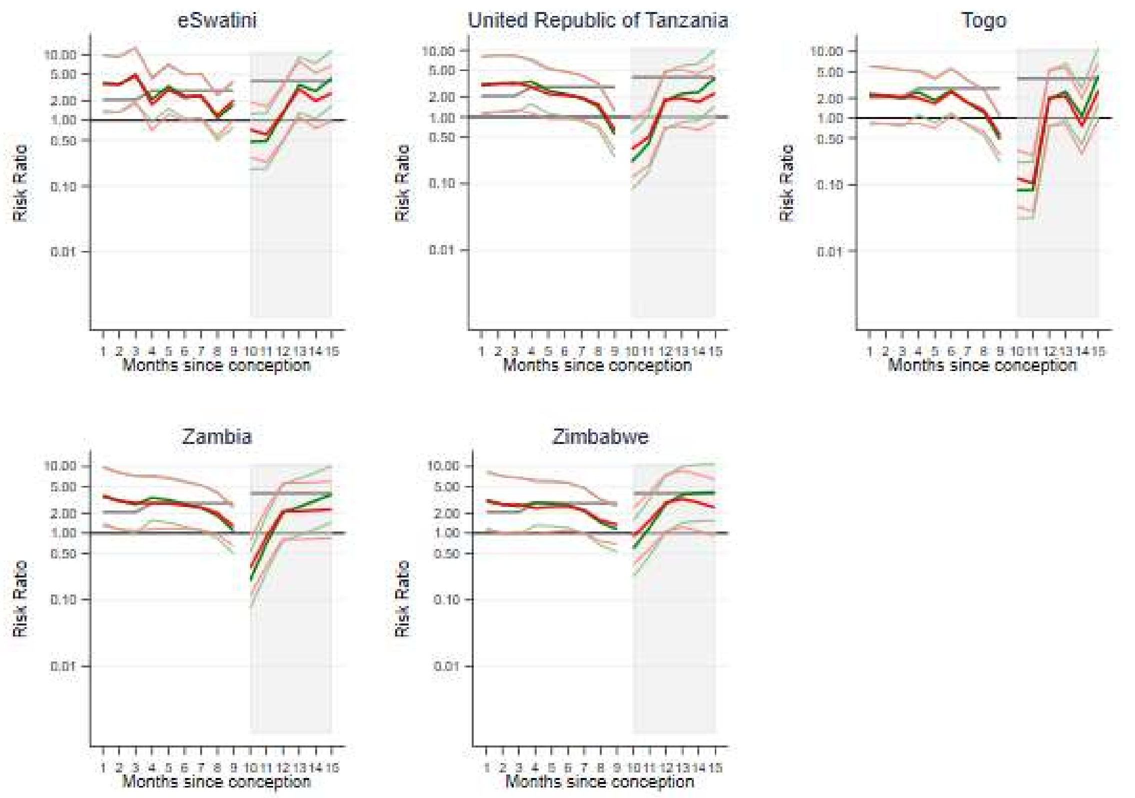

Figure S3: Estimated population level acute infection ratios, comparing the last month of pregnancy and the breastfeeding postpartum period to not pregnant not postpartum women Assuming an acute infection window of three months (blue line) and two months (red line). The grey line is the estimated acute infection ratio using direct per-condomless-coital-act HIV acquisition risk ratios with an assumption of a three month infection window.

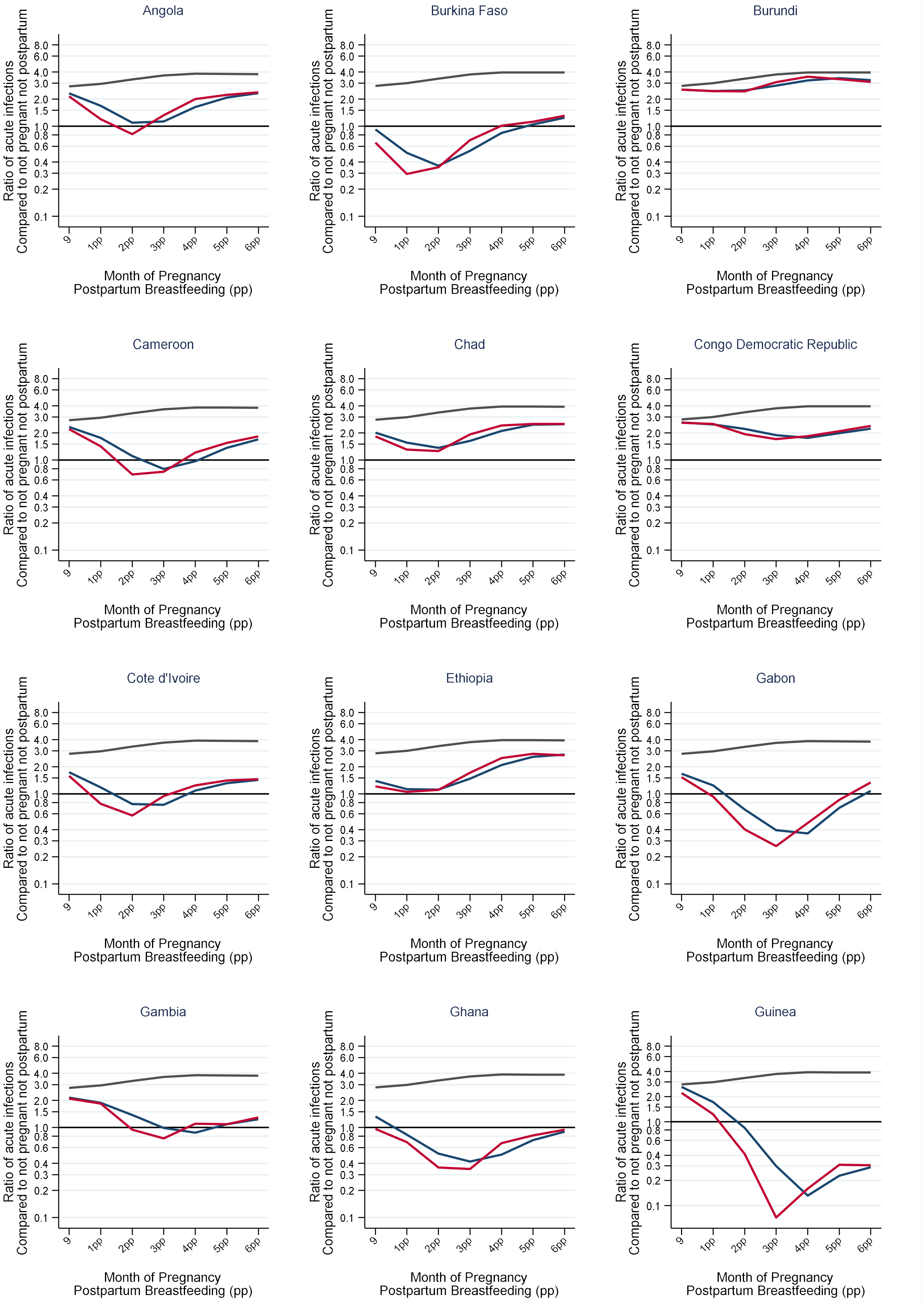

Figure S3 continued: Estimated population level acute infection ratios, comparing the last month of pregnancy and the breastfeeding postpartum period to not pregnant not postpartum women Assuming an acute infection window of three months (blue line) and two months (red line). The grey line is the estimated acute infection ratio using direct per-condomless-coital-act HIV acquisition risk ratios with an assumption of a three month infection window.

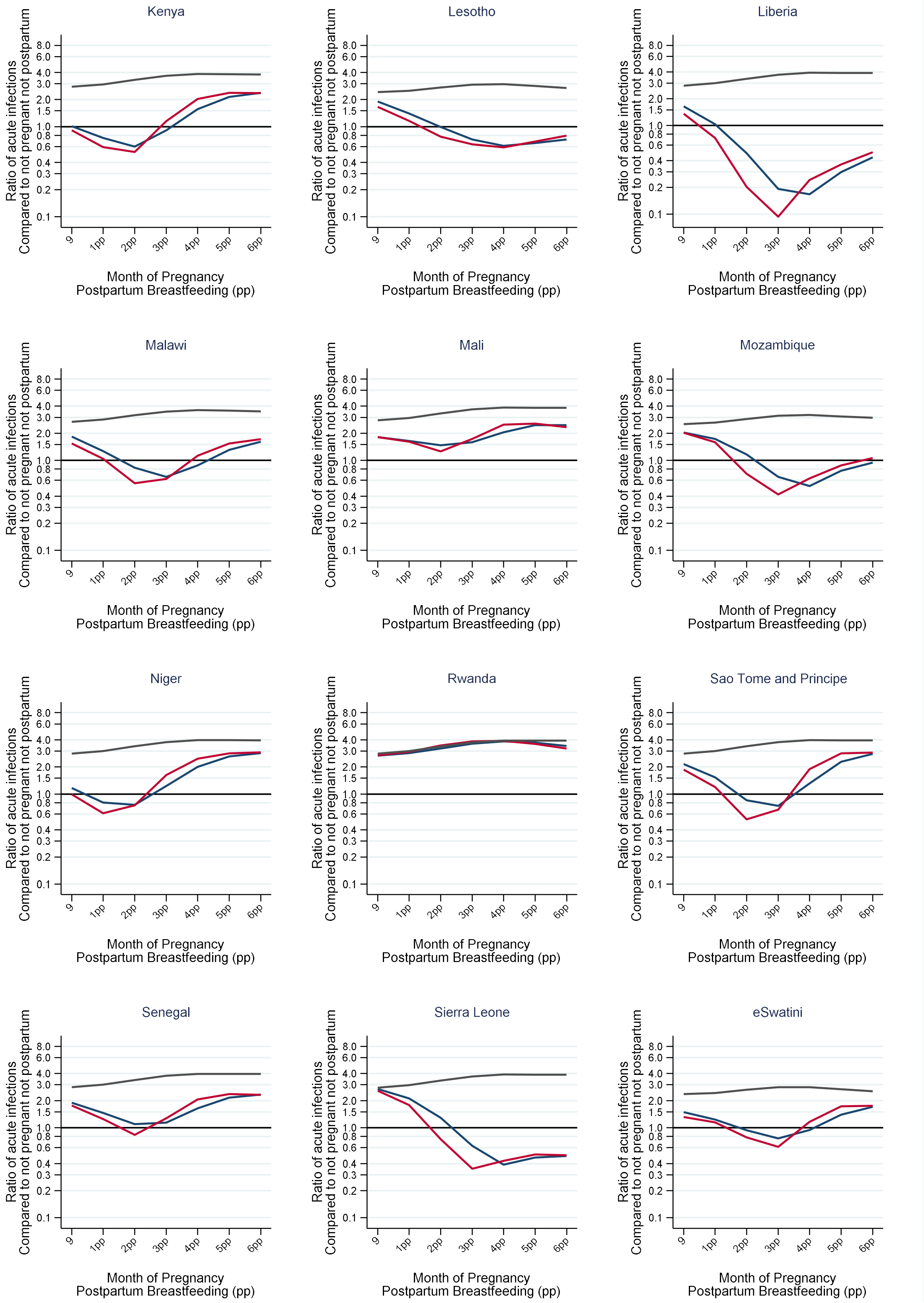

Figure S3 continued: Estimated population level acute infection ratios, comparing the last month of pregnancy and the breastfeeding postpartum period to not pregnant not postpartum women Assuming an acute infection window of three months (blue line) and two months (red line). The grey line is the estimated acute infection ratio using direct per-condomless-coital-act HIV acquisition risk ratios with an assumption of a three month infection window.

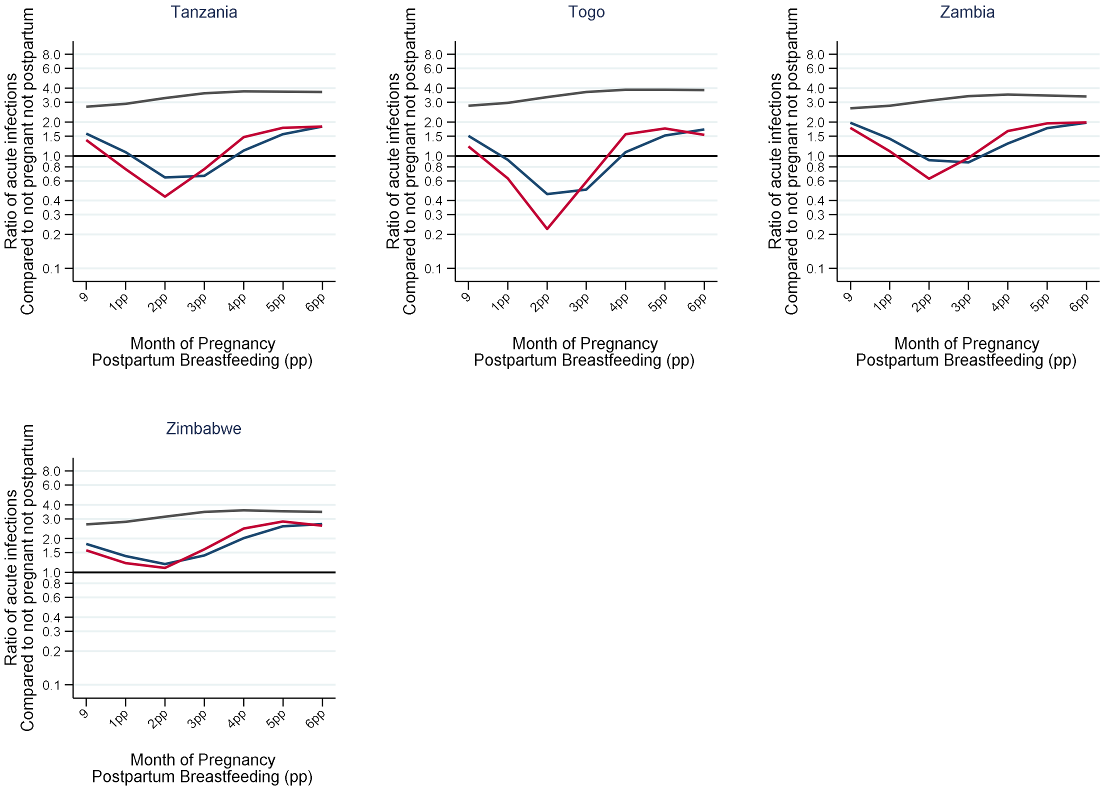

Figure S4: Estimated ratio of acute infections in the fourth to sixth month of pregnancy, compared to not pregnant/postpartum(pp) women, assuming no ART initiation. Estimates use per-condomless-coital-act estimates taking into account differences in sexual activity in the last week and HIV-serodiscordancy with a partner, assuming an acute infection window of three months. Shown for each country given their sexual activity patterns (blue). Confidence intervals represent uncertainty around the per-condomless-coital-act risk ratio estimates. Also plotted is the point estimate of the per-condomless-coital-act risk ratios from Thomson et al 2018 (red).

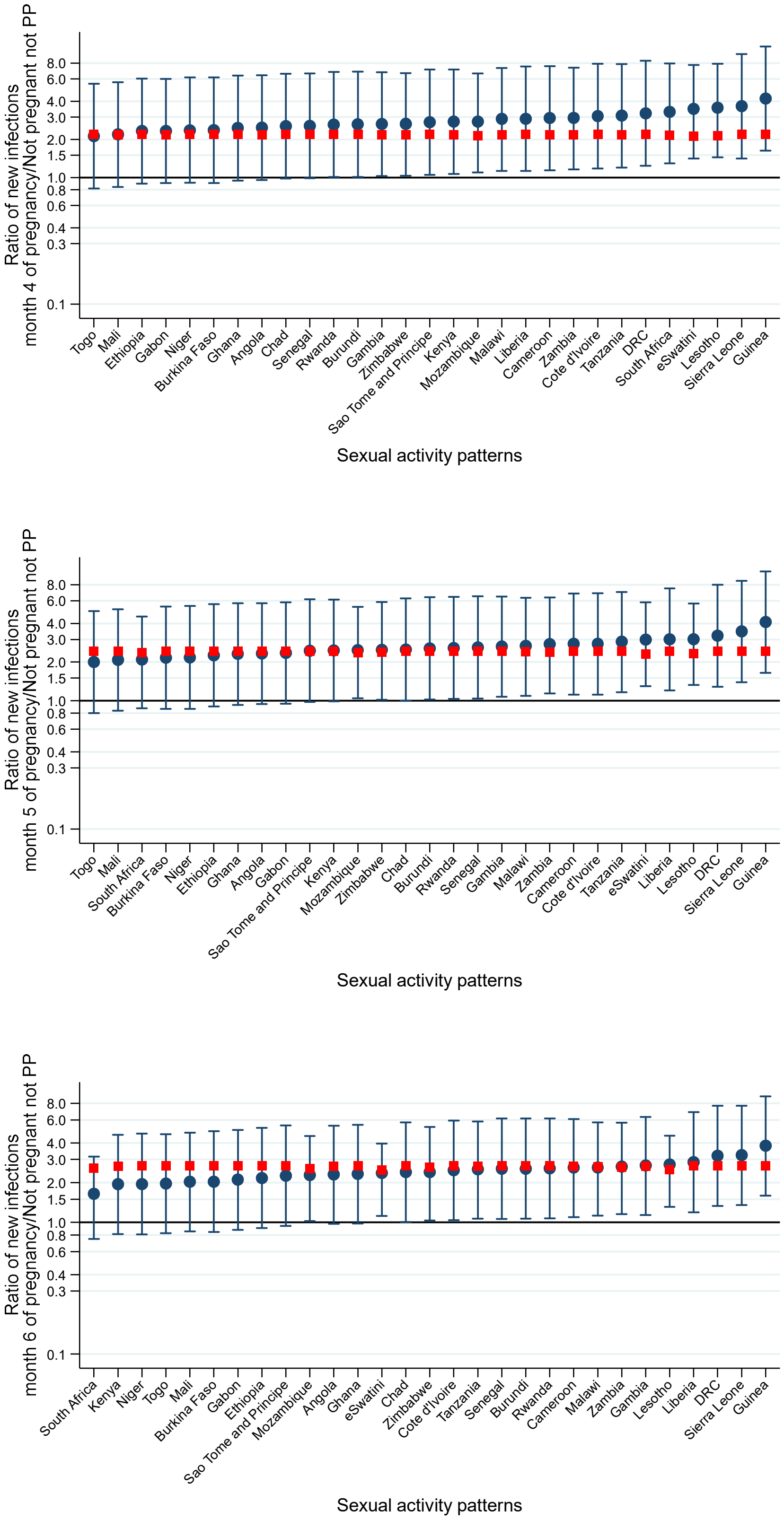

Figure S5: Estimated ratio of acute infections in the seventh to ninth month of pregnancy, compared to not pregnant/postpartum(pp) women, assuming no ART initiation. Estimates use per-condomless-coital-act estimates taking into account differences in sexual activity in the last week and HIV-serodiscordancy with a partner, assuming an acute infection window of three months. Shown for each country given their sexual activity patterns (blue). Confidence intervals represent uncertainty around the per-condomless-coital-act risk ratio estimates. Also plotted is the point estimate of the per-condomless-coital-act risk ratios from Thomson et al 2018 (red).

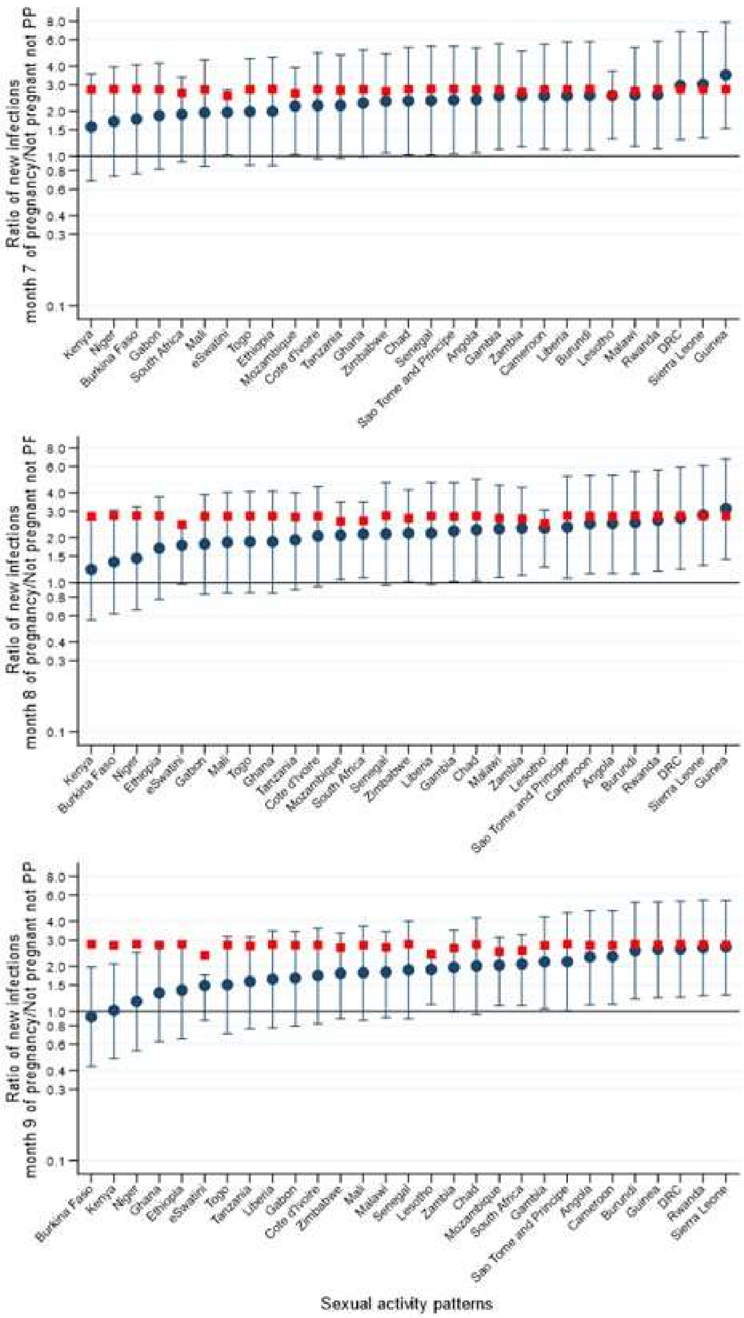

Figure S6: Estimated ratio of acute infections in the first to fourth month postpartum (pp), compared to not pregnant/postpartum women, assuming no ART initiation. Estimates use per-condomless-coital-act estimates taking into account differences in sexual activity in the last week and HIV-serodiscordancy with a partner, assuming an acute infection window of three months. Shown for each country given their sexual activity patterns (blue). Confidence intervals represent uncertainty around the per-condomless-coital-act risk ratio estimates. Also plotted is the point estimate of the per-condomless-coital-act risk ratios from Thomson et al 2018 (red).

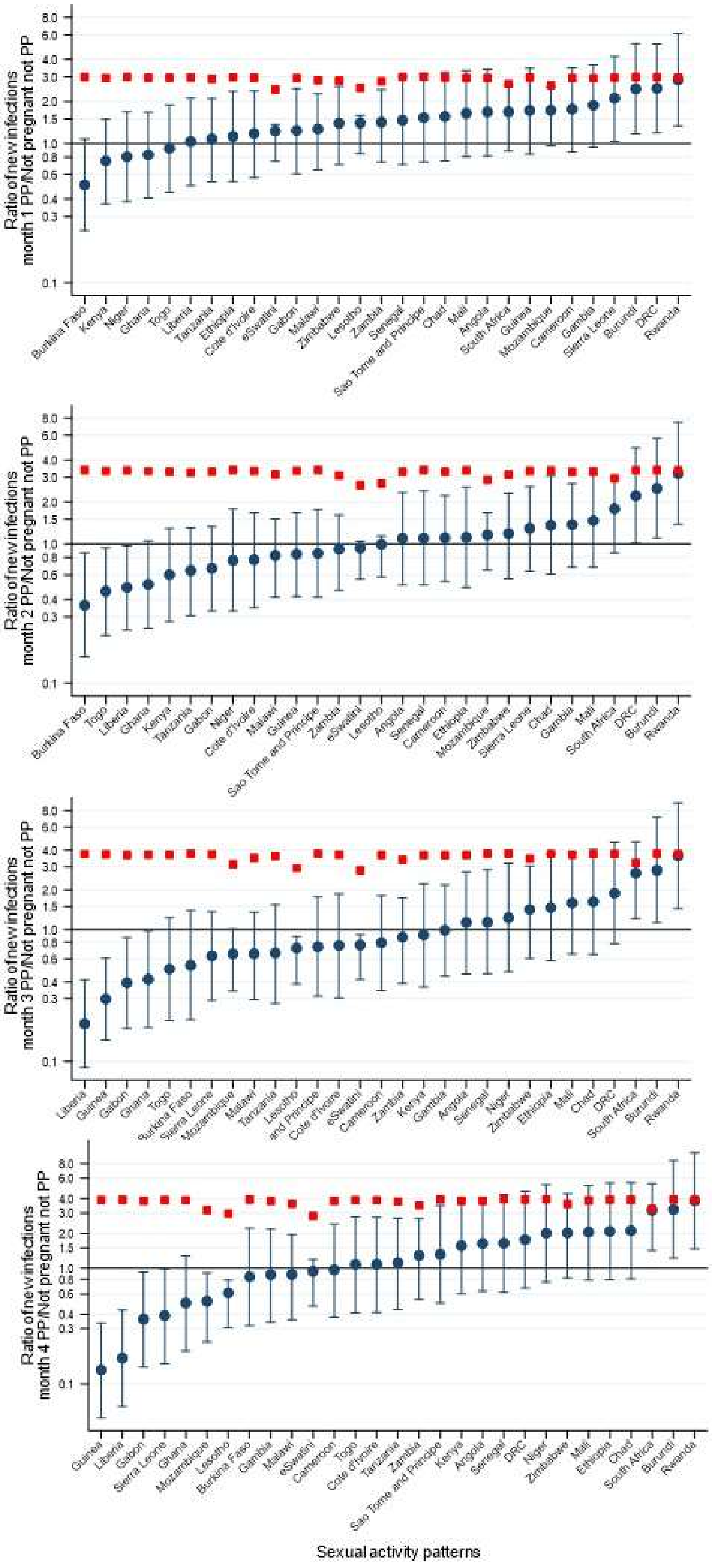

Further Analyses

Exploring reasons for differences in serodiscordancy - Methods

We hypothesised that the reason for differences in discordancy in HIV negative pregnant and postpartum women compared to those not pregnant/postpartum is that they are likely to have been exposed to their partner for longer or at higher frequency than those not pregnant/postpartum due to a) sexual activity being necessary to become pregnant and b) It is common to have children with the same partner, therefore pregnancies beyond the first will likely be with a partner to whom the women has been exposed.

We first looked at the relationship between duration of partnership and having an HIV infected partner for HIV negative women using the couple's dataset. We then looked at how the relationship between maternity statuses differs by duration of partnership. The DHS asks age at first marriage and calculates years since first marriage, therefore for those who have only had one union (identified in the DHS), we have the duration of the partnership, however for those currently in a union that is not their first we do not know the length of the partnership. Even in the partnership loop questions, where it is asked how long since your partnership started, if the partner is a spouse, the data is unavailable. Therefore, we restricted our analysis to HIV negative women who are still in their first union. We used marital duration first grouped into less than one year, one to two years, three to five years and five years and over. We then grouped it into a binary variable of less than one year or greater than one year. We use log binomial regression to calculate the risk ratios of being serodiscordant comparing pregnancy and postpartum women to those not pregnant/postpartum by duration of partnership, further adjusting for five-year age group, country and calendar year. We also calculated risk ratios comparing the risk of having an HIV positive partner with a marital duration of over one year compared to less than one year.

Results

The couple dataset used for the serodiscordancy analysis represents a varying proportion of all women by survey and by maternity status. For women not pregnant or postpartum the couple dataset represents between 24% to 72% of all women, for pregnant women it is much higher ranging between 39% to 93% and the postpartum period similar to pregnancy at between 29%-93%.

Using the data from cohabiting partners, HIV negative women who had only been married or cohabited once, those in their first year of the relationship were 1.37 (95%CI 1.07-1.74) times more likely to have an HIV positive infected partner compared to those in a relationship duration longer than a year when adjusting for five-year age group, place of residence, country and survey year. There was no evidence of a further decrease as duration of marriage increased beyond one year. There was no evidence that this effect varied by five-year age group (Wald test F=045, p=0.771).

The risk of an HIV negative women having an HIV positive partner comparing pregnant and postpartum women with those not pregnant or postpartum was consistently lower if the women are in their first year of marriage compared to being married for more than one year (Table S5).

Marital duration as defined as less than one year and more than one year was similar in the couple dataset when compared to the all women dataset, although there was a small decreased risk of being in a relationship for more than one year of 0.983 (95%CI 0.981-0.986) when adjusting for five-year age group, country and calendar year. There was no evidence for this varying over months since conception or five-year age group.

Table S5: Risk Ratio comparing the risk of an HIV negative woman in her first marriage having an HIV positive partner by pregnancy trimester and postpartum period compared to not pregnant/postpartum, by duration of the marriage.

	Risk of an HIV positive partner			
	Marital duration <1year		Marital duration >1year	
	RR	95%CI	RR	95%CI
Not pregnant/postpartum	1		1	
First trimester	0.74	(0.35-1.59)	0.95	(0.71-1.28)
Second trimester	0.64	(0.34-1.21)	0.79	(0.61-1.02)
Third trimester	0.42	(0.19-0.92)	0.67	(0.50-0.89)
Early postpartum	0.54	(0.21-1.41)	0.89	(0.69-1.14)
Late postpartum	0.51	(0.13-2.04)	1.06	(0.85-1.31)

Discussion

In this analysis we found that HIV negative women in their first marriage were more likely to have an HIV positive partner in the first year of marriage compared to later years. This is probably due to the fact that at later duration of marriage women have on average had the same exposure whether pregnant or not, as most will have been pregnant before, so those pregnant and postpartum are more similar to those who are not. However, in early marriage, pregnancy would indicate higher frequency of condomless sex therefore a higher chance of becoming concordant positive with a partner sooner than those not pregnant, causing greater differentials between the two groups. From this analysis it appears that among married couples, although longer duration of partnership means that an HIV negative woman is less likely to have an HIV positive partner, what differentiates levels of discordancy in HIV negative pregnant/postpartum women from those not pregnant/postpartum is higher levels of sexual activity prior to or during pregnancy.
